# GRK3 is a poor prognosticator and serves as a therapeutic target in advanced gastric adenocarcinoma

**DOI:** 10.1186/s13046-022-02463-6

**Published:** 2022-08-23

**Authors:** Yuan Li, Yibo Fan, Jinbang Xu, Longfei Huo, Ailing W. Scott, Jiankang Jin, Boxuan Yang, Shan Shao, Lang Ma, Ying Wang, Xiaodan Yao, Melissa Pool Pizzi, Matheus Sewastjanow Da Silva, Guoliang Zhang, Lijuan Zhuo, Eun Jeong Cho, Kevin N. Dalby, Namita D. Shanbhag, Zhenning Wang, Wenliang Li, Shumei Song, Jaffer A. Ajani

**Affiliations:** 1 Department of Gastrointestinal Medical Oncology, The University of Texas MD Anderson Cancer Center, 1515 Holcombe Blvd., Houston, TX 77030 USA; 2 Department of Surgical Oncology and General Surgery, First Hospital of China Medical University, Shenyang, 110001 People’s Republic of China; 3 Brown Foundation Institute of Molecular Medicine for the Prevention of Human Diseases, McGovern Medical School, The University of Texas Health Science Center, 1825 Pressler St., Houston, TX 77030 USA; 4 Fujian Maternity and Child Health Hospital, Affiliated Hospital of Fujian Medical University, Fuzhou, 350001 People’s Republic of China; 5 Division of Chemical Biology and Medicinal Chemistry, College of Pharmacy, The University of Texas at Austin, Austin, TX 78712 USA

**Keywords:** Gastric adenocarcinoma, GRK3, Hippo/YAP1, Prognosis, Peritoneal metastases

## Abstract

**Background:**

G protein-coupled receptor (GPCR) is the most targeted protein family by the FDA-approved drugs. GPCR-kinase 3 (GRK3) is critical for GPCR signaling. Our genomic analysis showed that GRK3 expression correlated with poor prognosis of gastric adenocarcinoma (GAC) patients. However, GRK3’s functions and clinical utility in GAC progression and metastases are unknown.

**Methods:**

We studied GRK3 expression in normal, primary, and metastatic GAC tissues. We identified a novel GRK3 inhibitor, LD2, through a chemical-library screen. Through genetic and pharmacologic modulations of GRK3, a series of functional and molecular studies were performed *in vitro* and *in vivo*. Impact of GRK3 on YAP1 and its targets was determined.

**Results:**

GRK3 was overexpressed in GAC tissues compared to normal and was even higher in peritoneal metastases. Overexpression (OE) of GRK3 was significantly associated with shorter survival. Upregulation of GRK3 in GAC cells increased cell invasion, colony formation, and proportion of ALDH1^**+**^ cells, while its downregulation reduced these attributes. Further, LD2 potently and specifically inhibited GRK3, but not GRK2, a very similar kinase to GRK3. LD2 highly suppressed GAC cells’ malignant phenotypes *in vitro*. Mechanistically, GRK3 upregulated YAP1 in GAC tissues and its transcriptional downstream targets: SOX9, Birc5, Cyr61 and CTGF. Knockdown (KD) YAP1 rescued the phenotypes of GRK3 OE in GAC cells. GRK3 OE significantly increased tumor growth but LD2 inhibited tumor growth in the PDX model and dramatically suppressed peritoneal metastases induced by GRK3 OE.

**Conclusions:**

GRK3, a poor prognosticator for survival, conferred aggressive phenotype. Genetic silencing of GRK3 or its inhibitor LD2 blunted GRK3-conferred malignant attributes, suggesting GRK3 as a novel therapeutic target in advanced GAC.

**Supplementary Information:**

The online version contains supplementary material available at 10.1186/s13046-022-02463-6.

## Background

Gastric adenocarcinoma (GAC) is the fifth most common cancer and the third leading cause of cancer-related mortality worldwide [1]. GAC is usually diagnosed in the late stages, and metastatic GAC is invariably incurable [2–4]. Despite some advances in therapies such as surgery, systemic therapy, and radiation therapy, GAC mortality rates remain high. Advanced genetic and molecular profiling has yielded a vast quantity of new information for potential therapeutic exploitation and risk stratification [5, 6]. However, novel predictive and prognostic biomarkers to serve as therapeutic targets are still limited.

Through shRNA and cDNA functional screening of human kinases, we previously identified that GRK3 (G protein-coupled receptor kinase 3) is an essential kinase for prostate cancer progression [7]. Yet, the mechanism of its actions in cancer progression is still largely unclear. GRK3 is best known to phosphorylate the agonist-occupied form of the β-adrenergic and related G protein-coupled receptors, leading to broad regulation of receptor functions. G protein-coupled receptors (GPCRs) play a central role in signal transmission, thereby controlling many facets of cellular functions. Increasing evidence suggests a role of GPCRs and their ligands in different aspects of tumor biology [8].

GRK3 shows varied expression levels and functions in different tumor types. For example, downregulation of GRK3 correlated with increased growth of glioblastoma cells [9] and was associated with worse outcomes in pancreatic ductal adenocarcinoma [10] and hepatocellular carcinoma [11]. In contrast, over-expression of GRK3 in prostate tumor cells and colon cancer promoted tumor growth and metastases through the induction of angiogenesis [7, 12]. Therefore, the roles of GRK3 in various cancers are likely context dependent. However, the role of GRK3 in GAC remains unclear and needs to be pursued.

In this study, we evaluated the expression of GRK3 in primary GAC tissues and paired adjacent normal tissues as well as in peritoneal carcinomatoses (PC) cells from GAC patients. We then elucidated the functional role of GRK3 in promoting tumor growth and metastases using genetic approaches. Mechanistically, we identified crosstalk between GRK3 and YAP1, a key player in GAC progression and metastases [13]. Importantly, we identified a novel GRK3 inhibitor, LD2, through a chemical library screen and demonstrated its strong anti-tumor activity *in vitro* and *in vivo* in patient-derived PC cells with high GRK3 and YAP1 expressions. Thus, GRK3 could serve as a prognostic biomarker and novel therapeutic target in GAC.

## Materials and methods

### Cells and reagents

The human GAC cell lines AGS, MKN45, SK-GT-5 (GT-5), KATO III, SNU-1, and SNU-16 were purchased from the American Type Culture Collection (ATCC, Manassas, Virginia) and previously described [14]. The immortalized normal gastric epithelial cell lines GES-1 and HFE145 were described previously [15, 16]. GA0518 patient-derived cells were isolated from a patient-derived xenograft (PDX) model implanted with PC cells from a GAC patient [14]. Patient-derived GA0804, GA0825, GA0515, and GA0313 cells were enriched directly from patient’s PC samples after lysing red blood cells. These cells were grown in RPMI 1640 medium with 10% fetal bovine serum at 37 °C in 5% CO_2_. All human cell lines were authenticated in the Cytogenetics and Cell Authentication Core facility of The University of Texas MD Anderson Cancer Center every 6 months and tested for mycoplasma in our laboratory every 6 months. Antibodies used included anti-GRK3 rabbit antibody (Abcam, Cambridge, United Kingdom), anti-YAP1 rabbit antibody (Cell Signaling Technology, Danvers, MA), anti-SOX9 rabbit antibody (EMD Millipore, Billerica, MA), anti-CTGF mouse antibody (Santa Cruz Biotechnology, Santa Cruz, CA), anti-Cyr61 rabbit antibody (Santa Cruz Biotechnology, Santa Cruz, CA), and anti-Survivin rabbit antibody (Cell Signaling Technology, Danvers, MA)**.** LD2 was identified by screening chemical libraries for inhibition of GRK3’s kinase activity, performed by Dr. Kevin Dalby’s laboratory at The University of Texas at Austin and Dr. Wenliang Li at The University of Texas Health Science Center at Houston. LD2 was provided by Dr. Wenliang Li for the experiments in this study.

### Patient samples and tumor tissue microarrays (TMAs)

Ethical approval for this study was obtained from the ethics committee of China Medical University (CMU) and the Institutional Review Board of MD Anderson Cancer Center (MDACC). All patients who volunteered to provide research specimens signed an approved written consent document. Experiments were carried out at CMU and MDACC on respective tissue resources available at each institution.

Twenty pairs of GAC and normal tissues were from the First Hospital of CMU, and 3 pairs of tissues as well as PC samples from 42 cases were from the Department of Gastrointestinal Medical Oncology at MDACC for quantitative real-time polymerase chain reaction (qPCR). The 42 PC samples for immunofluorescent staining were from the Department of Gastrointestinal Medical Oncology at MDACC. The TMA included a total of 422 patients who underwent total or subtotal gastrectomy with lymphadenectomy for GAC from January 2009 through December 2014 from the Department of Surgical Oncology and General Surgery at First Hospital of CMU. Among 422 patients, 393 patients have complete clinical survival outcomes that were analyzed for survival association for GRK3 expression. Written informed consents were obtained from all patients. None of the patients had received chemotherapy or radiotherapy before their surgical procedure. Detailed postoperative pathology reports and other demographic and clinical data were obtained, including age, gender, tumor location, tumor size, differentiation status, growth pattern, invasion depth, lymph node metastasis, distant metastases, TNM stage, and vein invasion. We used the TNM classification for GAC based on the 7th edition of the AJCC staging manual. All patients were followed up via telephone inquiries or questionnaires. The follow-up time was 2–98 months (median 51 months).

### Immunohistochemistry

For formalin-fixed, paraffin-embedded TMA, 5-μm-thick tissue sections were deparaffinized in xylene, followed by dehydration in an ethanol series. The slides were subjected to antigen retrieval with 10 mM sodium citrate (pH 6.0) for 45 min. Before staining, non-specific binding was blocked by incubation with hydrogen peroxide as a peroxidase suppressor (Thermo Fisher Scientific, Waltham, MA) and normal horse serum (Vector Laboratories, Burlingame, CA) as a blocking buffer, followed by incubation with 1:100 anti-GRK3 (ab109303, Abcam) antibodies in antibody diluent (BioGenex, San Ramon, CA) at 4 °C overnight. All sections were briefly washed with PBS and incubated at room temperature with horseradish peroxidase-conjugated secondary anti-rabbit antibody. The color was then developed by incubation with a DAB substrate kit (Vector Laboratories). Nuclei were counterstained blue with hematoxylin (Sigma–Aldrich, St. Louis, MO) and mounted in VectaMount Permanent mounting medium (Vector Laboratories). Isotype immunoglobulin G controls were used as negative controls for the staining.

Two pathologists who were blinded to patient outcomes independently interpreted the immunostaining results using a semi-quantitative scoring system. Immunostaining reactions were evaluated by staining intensity (0, no staining; 1, weak staining; 2, moderate staining; and 3, strong staining) and the percentage of stained cells (0, ≤ 5%; 1, 5–25%; 2, 25–50%; 3, 50–75%; 4, > 75%). Then, the percentage of positive cells and the staining intensity were multiplied to generate the immunoreactivity score (IS) for each case. If there were discrepancies in the IS as determined by the two pathologists, specimens were rescored until a consensus was reached. Then the cutoff value was defined by the ROC curve. The cutoff value of IS was 1, so the IS = 0 was defined as negative and IS > = 1 as positive.

### Indirect immunofluorescence

PC cells were used to produce cell blocks for slides, and some paired primary tumor tissues were subjected to indirect immunofluorescence staining with anti-GRK3 (1:100), anti-SOX9 (1:2000), anti-CTGF (1:100), anti-Cyr61 (1:100), and anti-Survivin (1:100) then labeling with Alexa Fluor 555 (for GRK3, Cyr61, and Survivin) and Alex Fluor 488 (for SOX9 and CTGF) as described previously [17, 18]. Fluorescence was observed on a confocal microscope (FluoView FV500; Olympus America, Melville, NY) and analyzed by CellQuest Pro software (BD Biosciences, Franklin Lakes, NJ).

### Generation of GAC cells with genetic knockdown or overexpression of GRK3

GRK3 gene knockdown (KD) by the lentiviral CRISPR (Clustered Regularly Interspaced Short Palindromic Repeats) system was performed following instructions on the Massachusetts Institute of Technology website (http://crispr.mit.edu/, which is discontinued now). A second software for designing guide RNAs (gRNAs) was obtained from the German Cancer Research Center (http://www.e-crisp.org/E-CRISP/designcrispr.html) gRNA design guidelines. The designed pairs of gRNA (Suppl Table 2) formed duplexes which were then ligated using T4 ligase to V2mO (pLenticrispr-V2-mOrange) (#140,206, Addgene, Cambridge, MA) precut by BsmB1 and gel purified. The ligates were transformed into Stbl3 competent cells. Clones on LB plates were screened, and the positive ones were verified by sequencing. The plasmids with desired gRNAs were then packaged into lentiviruses using psPax2 and pDM2.G at a ratio of 10:10:1 in HEK293T cells. Lentiviruses were transduced into desired target cell lines by infecting with polybrene at 8 μg/ml. Stable cell lines were obtained upon selection with puromycin at concentrations determined by its kill curves in different cell lines. The efficacy of gene KD in target cell lines was determined by Western blots and qPCR for further experiments.

GRK3 cDNA overexpression, constitutive shRNA silencing and inducible shRNA silencing were achieved by viral transduction and antibiotics selection, similarly to what we previously described [7]. Briefly, GRK3 wild type (WT) or kinase-dead mutant cDNA were in pJP1653 retroviral vector (blasticidin selection). Constitutive shGRK3 and control scramble shRNA were in pLKO-puro lentiviral vector (puromycine selection). Doxycycline-inducible shGRK3 and control scramble shRNA were in pCW39-neo vector (an inducible vector similar to Addgene's pLKO-Tet-On, G418 selection). Doxycycline at 0.2–0.5 μg/ml for 3–4 days was employed to inducibly expressing shGRK3. The rescue experiment in KATO III and MKN45 GRK3 OE cells by KD YAP1 using LentiCRISPR/CAS9 technology. Three pair of YAP1 guide RNA followed German Cancer Research Center (http://www.e-crisp.org/E-CRISP/designcrispr.html) guide RNA (gRNAs) design guidelines (Supplementary Table 1). The designed pairs of gRNA were T4 ligated to the pLentiCRISPR V2mOrange (V2mO, # 140,206, Addgene, Cambridge, MA). Clones are sequencing verified. Then, these KD virus from 293 T cells were transduced into the KATOIII and MKN45 GRK3 OE cells to generate YAP1 KD cells in GRK3 OE in both KATOIII and MKN45 cells to be able to perform the rescue experiments.

### qRT-PCR

Total RNA was extracted from GAC cells treated with LD2 at indicated dosage using Direct-zol RNA Kit (Zymo Research), processed for cDNA synthesis using the Reverse Transcription Kit (Applied Biosystems), and subjected to the qRT-PCR using SYBR Green Master Mix (Applied Biosystems). The expression level of indicated genes was normalized to the expression of *GAPDH* as housekeeping gene. Primer sequence were listed in Suppl Table 3.

### Matrigel invasion assay

The invasion assay was performed as described previously [19]. Briefly, 1 × 10^5^ cells of MKN45 or KATO III with overexpression of GRK3 compared with control or GA0518, GA0814 and AGS cells were plated on the well of transwell insert (Corning) and then 600 µl of culture medium with 20% FBS was gently added to the lower well followed by incubation of the cells with or without treated with LD2 at indicated dosage in 60 µl of culture medium containing 0.5% FBS at 37 °C, 5% CO2 for 48 h. The culture media and cells inside of the insert were removed with cotton-tipped applicators and the migrated cells were fixed with 4% formaldehyde and stained with 0.2% crystal violet. The migrated cells were counted under an invert microscope.

### Tumor sphere formation assay

GA0518, GA0804 and AGS cells (800/well) were seeded in triplicate onto a 24-well ultra-low attachment plate (Corning, Corning, NY) in serum-free DMEM /F-12 supplemented with 10 ng/ml epidermal growth factor, 5 mg/ml insulin, 0.5 mg/ml hydrocortisone, and bovine pituitary extract (Invitrogen). The cells were then treated with control DMSO or LD2 at the indicated doses. After 10–14 days of culture, tumor spheres formed (diameter > 100 μm) were counted under a microscope.

### Flow cytometry ALDH1^+^ labeling

ALDH1 activity was assessed by flow cytometry using an ALDEFLUOR kit (STEMCELL Technologies, Vancouver, Canada) per the manufacturer’s instructions. Cultured human GAC cells and PC cells were suspended in ALDEFLUOR assay buffer at 1 × 10^6^ cells/ml and added to a tube containing 5 μl of the ALDH1 substrate. As a negative control, a 0.5-ml aliquot from each sample was treated with ALDH1-specific inhibitor. After 30 min of incubation at 37 °C and then centrifugation, the cells were washed with the assay buffer, followed by resuspension in 0.3 ml of ice-cold ALDEFLUOR assay buffer for flow labeling (FACS Calibur, BD Biosciences).

### *In vivo* xenografts and PDX

*In vivo* experiments were conducted in accordance with the Institutional Animal Care and Use Committee. MKN45 GAC cells with GRK3 overexpression (GRK3 OE) and GFP control cells (NC) were subcutaneously injected into severe combined immunodeficient (SCID) mice (*n* = 5/group) and then monitored tumor growth for three weeks. After three weeks, tumor burden, tumor weight and tumor volume were measured as previously [13]. To determine the antitumor activity of LD2 in vivo, we applied a PDX model using GA0518 patient-derived cells., 5 × 10^6^ GA0518 cells were subcutaneously injected into severe combined immunodeficient (SCID) mice (*n* = 5/group, two injections per mouse on both flanks). After 7–10 days, the mice were injected with vehicle control DMSO: PBS (1:1) (100 μl/mouse) or LD2 (20 mg/kg) 5 times per week for 2 weeks. Tumor size was measured with a digital caliper (VWR International, Radnor, PA) once tumors reached a visible size, and tumor volume in mm^3^ was determined by the formula [(length × width)^2^] × 0.52, with length and width in mm. Mouse tumor weight and body weight were measured as described previously [20]. For the PC metastasis model, SCID mice were peritoneally injected with 1 × 10^6^ MKN45 GFP, MKN45 GRK3 OE, MKN45 GRK3 OE treated with LD2. 1 week after tumor cell injection, mice were randomly grouped. LD2 (20 mg/kg) was treated 5 times per week for 2 weeks. The tumor luminescent density was monitored every week. All these measurements were compared using the unpaired Student *t*-test.

### Statistical analysis

Student *t*-test and Fisher exact test were used to analyze colony formation and cell migration assays. Kaplan–Meier method was used to estimate the probability of survival. Log-rank test and Cox regression analysis were used to determine the association between markers and survival outcomes using SPSS 22.0 (IBM, Armonk, NY). Other assays are presented in graphs as mean ± standard error of the mean and represent the results of at least 3 experiments. The significance of differences between groups was judged using the 2-tailed Student *t*-test. Results were considered statistically significant if the P-value was less than 0.05. All statistical tests were performed using GraphPad Prism 8 software (GraphPad Software, Inc., San Diego, CA).

## Results

### Expression of GRK3 was increased in primary and metastatic GACs and associated with aggressive phenotype, and shorter survival

**Fig. 1:**
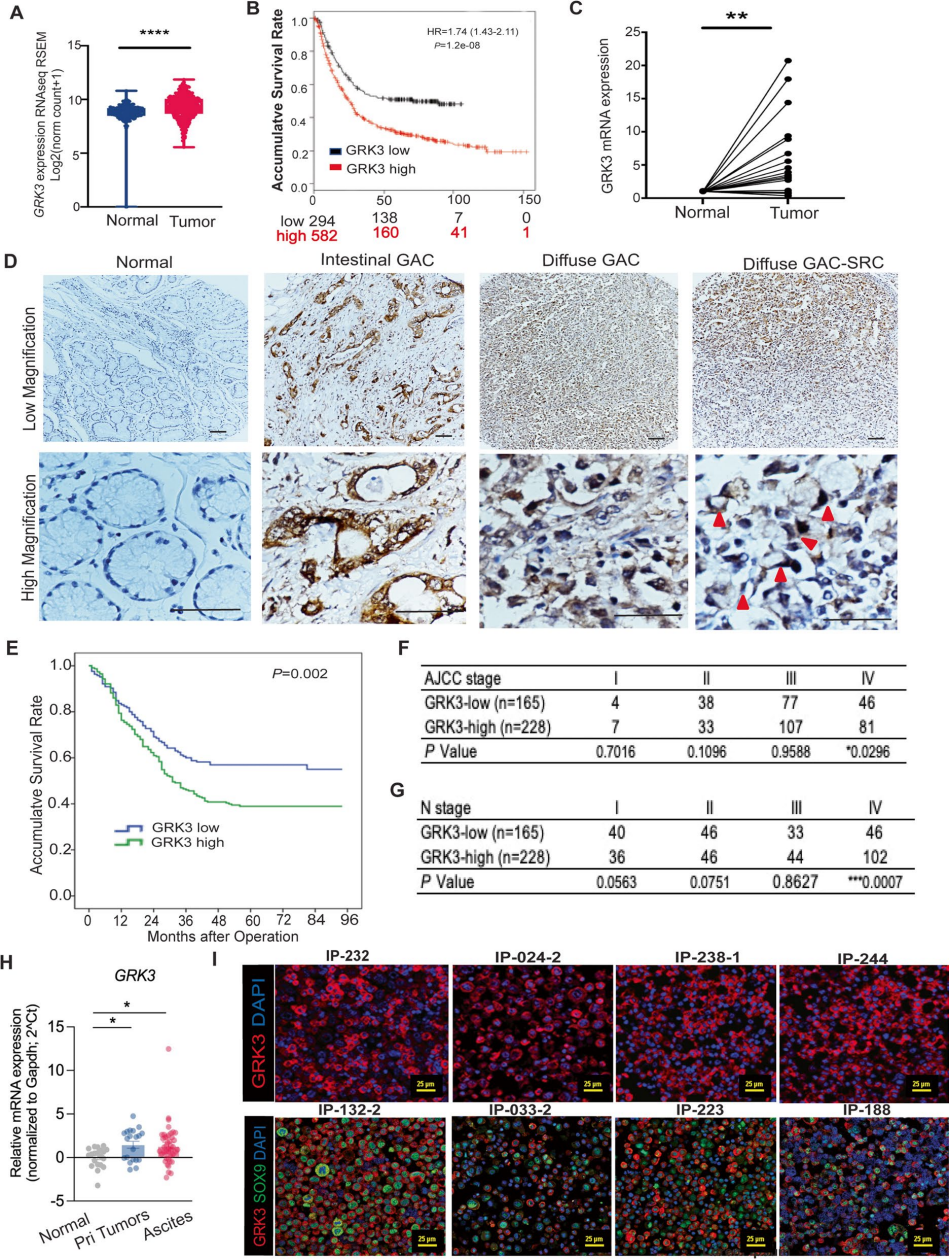
The expression of GRK3 is upregulated in primary and metastatic GACs, which is associated with aggressive phenotypes and shorter survival. **A** Expression of GRK3 is shown in GAC tumor tissues compared with normal tissues from TCGA STAD and GTEX cohort (****P < 0.0001). **B** Expression of GRK3 is associated with poor survival of 876 GAC patients in KM plotter database (*P* = 1.2e-08). **C** Expression of GRK3 mRNA was measured by quantitative polymerase chain reaction (qPCR) and normalized to GAPDH in 23 pairs of primary GAC tissues and adjacent normal tissues. (**P < 0.001). **D** Immunohistochemistry (IHC) staining was performed using the GRK3 antibody in GAC tissue microarrays (TMAs) of primary tumor tissues and adjacent normal tissues. **E** Kaplan–Meier analysis of overall survival time according to GRK3 expression level in GAC TMAs. GAC patients with high GRK3 expression showed worse survival than patients with low expression did (*P* = 0.002). **F**&**G** The expression of GRK3 was analyzed with stage and lymph node metastases in our GAC cohort in TMAs. **H** GRK3 mRNA level was detected on normal tissues, primary tissues, and ascites cells by qPCR. **P < 0.001. **I.** GRK3 expression was detected using immunofluorescent staining in 22 cases of malignant ascites cells. GRK3/DAPI staining are shown from 4 representative PC samples with high GRK3 (upper panel); Dual staining of GRK3 (Alexa Fluor 555, red) and SOX9 (Alexa Fluor 488, green) are shown in another 4 representative PC samples with high GRK3 (bottom panel). Scale bar: 25µm

To examine the expression status of GRK3 in GAC, we first explored TCGA dataset and found that expression of GRK3 was higher in gastric tumor tissues than that in normal tissues (Fig. 1A). Expression of GRK3 in tumor tissues in a large GAC cohort (*n* = 876) significantly associated with poor survival as well as a higher risk of death (*P* = 1.2e-08; hazard ratio = 1.74, confidence interval 1.43–2.11, kmplot.com) (Fig. 1B). To confirm these findings, we performed qPCR for GRK3 in 23 pairs of GAC tissues and adjacent normal tissues. Primers of qPCR for detecting GRK3 were listed in Supplementary Table 2. In 15 of 23 pairs (65%), GRK3 expression was higher in GAC tissues than in the adjacent normal tissues (Fig. 1C). To further validate this observation, we performed immunohistochemical (IHC) staining on a GAC TMA containing 393 pairs of GAC and normal tissues. GRK3 was typically expressed in the cytosol and cell membrane (Fig. 1D). Compared with the paired normal tissues, GRK3 expression was higher in GAC tissues. Both GAC phenotypes (intestinal and diffuse) and signet ring cell type of diffused GAC were positive for GRK3 (red arrow) (Fig. 1D). The positive expression rate for GRK3 was 61.1% (240/393) in GACs, but only 32.3% (127/393) in normal tissues. Intriguingly, GRK3 expression in the primary GACs was statistically associated with the higher rate of lymph node metastases, diffused type of GAC, and a higher TNM categories (Supplementary Table 3). The Kaplan–Meier analysis showed that GAC patients with higher GRK3 expression had shorter overall survival (*P* = 0.002, Fig. 1E). Univariate analysis found that GRK3 positive expression was significantly associated with high risk of death with HR = 1.563 and 95% confidence interval (CI) between 1.176–2.079 and *P* value was 0.002, although the multivariate analyses found that GRK3 is not an independent prognostic factor for survival (Supplementary Table 4). On further analysis, overexpression of GRK3 was associated with a higher AJCC stage and aggressive phenotype (Fig. 1F and G).

### GRK3 is highly expressed in peritoneal carcinoma (PC) cells from metastatic GAC patients

Based on the results in TMAs from our GAC cohorts, where GRK3 over-expression correlated with aggressive phenotype and lymph node metastases (Fig. 1G), we sought to determine the expression patterns of GRK3 in PC samples of GAC. First, we subcutaneously injected several patients’ PC cells (malignant ascites cells) into nude mice to generate PDXs as shown in Fig. S1A. Some PC cells (GA0518) formed tumors at the primary injection site (the primary tumor), and some mice further developed PC with ascites. We collected paired primary tumor cells and PC cells from the same mouse and determined GRK3 expression using Western blotting. GRK3 expression was higher in 3 PC samples compared with the paired primary tumors (Supplementary Fig. 1A, right panel). To confirm that the expression of GRK3 is higher in PC cells, we performed qPCR on primary tumors, normal adjacent tissues, and PC samples from 28 GAC patients. We found that GRK3 mRNA levels were upregulated in the primary tumors (*P* = 0.0042) and PC cells (*P* < 0.001, Fig. 1H) compared with adjacent normal tissues. Using immunofluorescence (IF) staining for GRK3 in PC cells from more advanced GAC patients, we found that 20 of 22 samples (90.9%) had high GRK3 expression, while only 2 samples showed weak expression. IF staining for GRK3 alone and dual staining of GRK3/SOX9 in representative PC samples are shown in Fig. 1I and Supplementary Fig. 1B. Furthermore, in 4 pairs of the primary tumors and PC cells, each pair from the same patient, GRK3 expression was higher in 3 PC samples (75%) than in the corresponding primary tumor (Supplementary Fig. 1C). Altogether, these results suggest that the expression of GRK3 is increased in PC cells and may play an important role in GAC progression and metastases.

### Upregulation of GRK3 in GAC cells augmented GAC cell growth and invasion, while downregulation of GRK3 inhibited malignant behavior of tumor cells *in vitro*

**Fig. 2:**
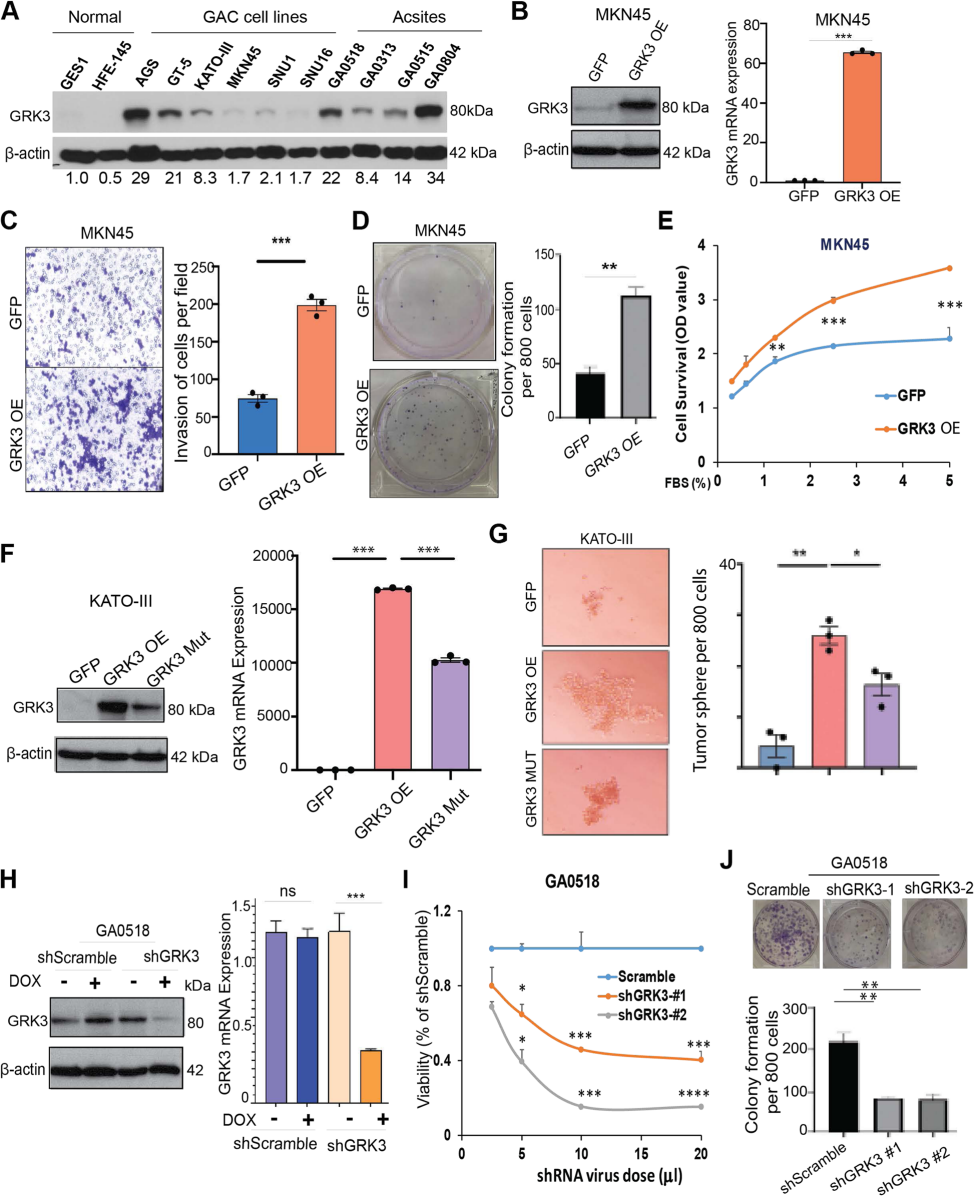
Upregulation of GRK3 increased GAC cell growth and invasion, while downregulation of GRK3 inhibited malignant behaviors of tumor cells *in vitro.* **A** GRK3 expression levels in normal gastric epithelial cell lines (GES-1 and HFE145), GAC cell lines (AGS, MKN45, GT-5, KATO III, SNU-1, and SNU-16), and patient-derived ascites cells (GA0518, GA0804, GA0515, and GA0313) were determined by western blot. Quantification of fold change for each cell line relative to GES-1 normal gastric cells and β-actin level was performed using Image J. **B** GRK3 was overexpressed in MKN45 cells by transducing GRK3 cDNA and confirmed by western blot (left) and qPCR (right). **C** Invasion assay was performed in MKN45-GRK3 overexpression cells compared to that of MKN45-GFP control cells; *** *P* < 0.0001. **D** Colony formation assay was performed in MKN45-GRK3 overexpression cells compared to that of MKN45-GFP control cells. **E** MKN45-GFP control and MKN45-GRK3 OE cells were cultured in media with a series of FBS % for 6 days. Cell survival and proliferation were measured by alamar blue viability assay. x-axis represents the series of FBS % in the culturing media. Values on y-axis are fold changes of growth for each % FBS relative to 0% FBS in either cell line. P values were calculated using the 2-sided Student’s t-test. ***: *P* < 0.005; **: *P* < 0.01. **F** GRK3 was overexpressed in KATO III cells by transfecting wild type (WT; GRK3 OE) or kinase-dead mutant form of GRK3 cDNA and confirmed by western blotting (left) and qPCR (right). **G** Tumor sphere formation assay was performed in KATO III GRK3 OE or mutant cDNA compared to that of KATO III-GFP control cells and quantification was quantified by image J analyses. *P < 0.01; ** *P* < 0.001. **H** GRK3 was KD in GA0518 PC cells using doxycycline inducible system and validated using western blot (left) and qPCR (right). **I.** GA0518 cells seeded in a 96-well plate were infected with a series of shRNA virus doses of shGRK3 #1 and shGRK3#2 and shScramble, from 20ul to 2.5ul per well. Cell survival and proliferation were measured by alamar blue viability assay 7 days after infection. X-axis represents the series of shRNA virus doses in the infections. Values on Y-axis are relative growth which was normalized to the scramble control shRNA for each virus dose for either cell line. P values were calculated using 2-sided Student’s t-test. ****: *P* < 0.0001; ***: *P* < 0.005; *: *P* < 0.05. **J**. Colony formation was determined in GA0518 cells with 2 independently GRK3 knockdown clones compared with control. **P < 0.001

Next, we investigated the functional role of GRK3 in GACs using genetic modulations. First, we assessed GRK3 expression in the indicated cell lines using western blotting (Fig. 2A). We found that protein levels of GRK3 in 6 GAC cell lines and 4 patient-derived PC cells (from malignant ascites) were much higher than in 2 normal gastric epithelial cell lines (Fig. 2A). Then, we overexpressed GRK3 by transfected GRK3 cDNA into MKN45 cells with low GRK3 and confirmed the successful transfection of GRK3 in MKN45 cells using western blot and q-PCR (Fig. 2B). We found tumor cell invasion and colony formation were significantly increased in GRK3 overexpressed MKN45 cells compared to MKN45-GFP control cells (Fig. 2C and D). Further, overexpression of GRK3 in MKN45 cells significantly increased tumor cell growth at any FBS concentration used (0–5%) media in 6 day (Fig. 2E) and (Supplementary Fig. 2A) and increased tumor sphere formation (Supplementary Fig. 2B). Similarly, overexpression of GRK3 wild type cDNA in KATO III cells significantly increased its tumor sphere formation, invasion, colony formation and proliferation, which was reduced in its kinase dead mutant form (Fig. 2F, G and Supplementary Figs.2C, 2D and 2E). In contrast, Knockdown (KD) of GRK3 in GA0518 patient-derived cells with relatively high GRK3 level (Fig. 2H) significantly decreased cell growth in two individual KD clones and reduced colony formation and tumor sphere compared to control cells (Fig.2I, J and Supplementary Fig. 2F). Similarly, KD of GRK3 in AGS cells with relatively high GRK3 level significantly decreased cell growth in two individual KD clones (Supplementary Fig. 2G). Altogether, these data suggest that GRK3 plays an oncogenic role in GAC.

### Identification of a GRK3 inhibitor LD2 that strongly suppressed the malignant behavior of GAC cells *in vitro*

**Fig. 3:**
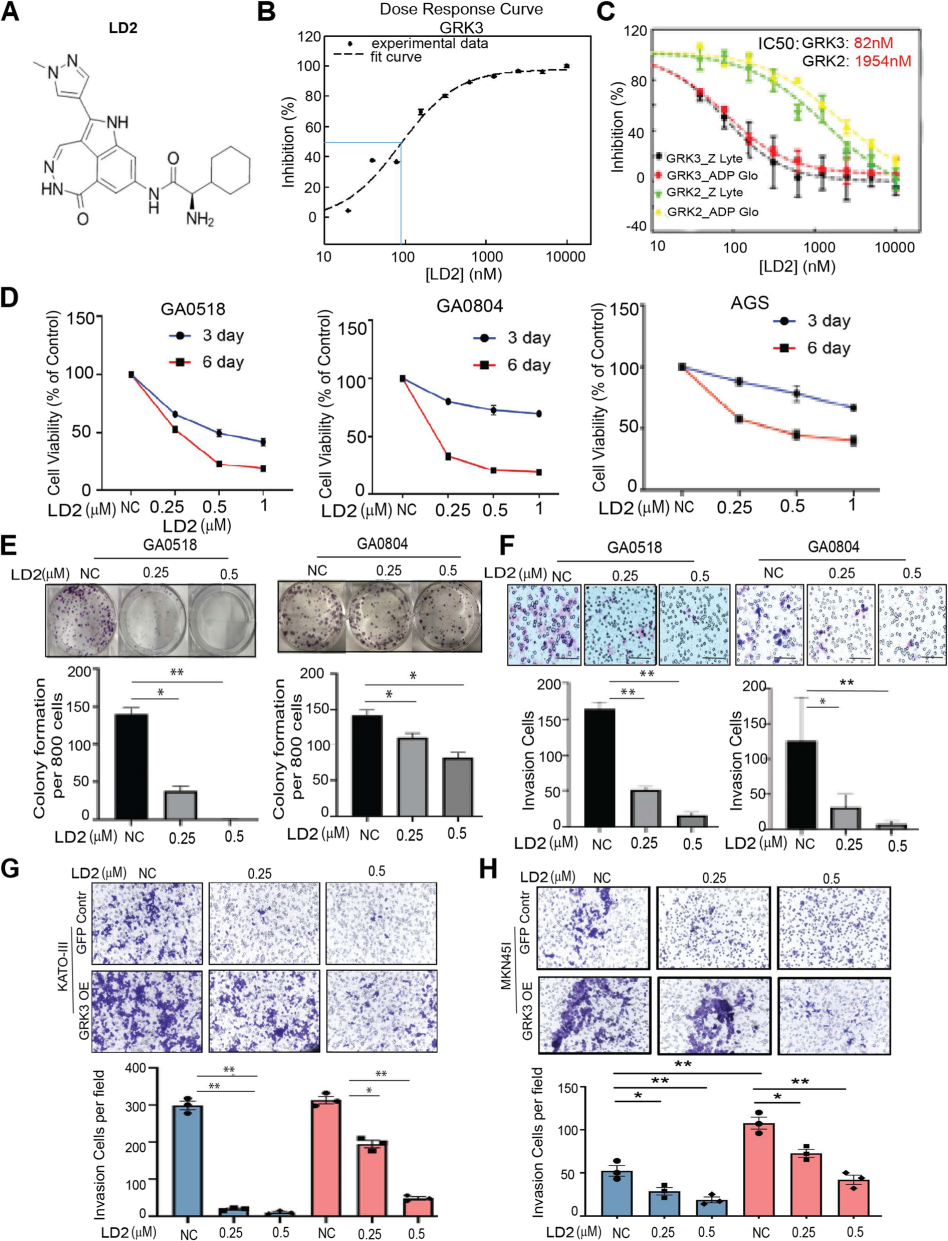
Identification of a novel GRK3 inhibitor LD2 that strongly suppressed the aggressive phenotypes of GAC cells *in vitro.* **A** The chemical structure of LD2 was shown. **B**&**C** The dose curves of LD2 effects in reducing kinase activities of GRK3 and its closest kinase GRK2. Two types of in vitro kinase assays were carried out to determine IC50 values for LD2 inhibition of GRK3 and GRK2: Invitrogen Z-LYTE and Promega ADP-Glo kinase assay. Plotted on y-axis is the % of remaining kinase activities of GRK3 and GRK2 upon treating with a series of LD2 doses. **D** LD2 suppressed tumor cell growth in GA0518, GA0804 and AGS cells treated with LD2 at the dosage as indicated. Cell survival was detected by MTS assay at 3 day and 6 day respectively. **E**& **F** LD2 suppressed colony formation (**E**), and cell invasion (**F**) in a dose-dependent manner. *P < 0.01; **P < 0.001. **G** Invasion assay was performed in KATO III-overexpression of GRK3 compared to that of KATO III-GFP control cells, and then treated with LD2 in indicated dosage for 48 h. Images of invasion (upper panel) and quantification (bottom panel) were shown. *P < 0.01; ** *P* < 0.001. **H** Invasion assay was performed in MKN45-overexpression of GRK3 cells compared to that of MKN45-GFP control cells, and then treated with LD2 in indicated dosage for 48 h. Images of invasion (upper panel) and quantification (bottom panel) were shown. *P < 0.01; ** *P* < 0.001

Since GRK3 belongs to the kinase family, which is known to be druggable [21, 22], we elected to investigate the potential of targeting GRK3 with inhibitors to block GAC progression. To this end, we screened a library of compounds for inhibiting GRK3 kinase using recombinant GRK3 protein and the Z-LYTE *in vitro* kinase assay from Invitrogen. Flow charts of our compound library screening strategies for identifying novel GRK3 inhibitors is shown in Supplementary Fig. 3. The primary screen was carried out using the Invitrogen Z-LYTE assay with single dose of 10 µM of compounds in the library. After the primary screen, hits were cherry picked and assayed for a dose response curve with 9 compound dilutions (twofold serial dilution) (separate manuscript in preparation). From these screens, we identified a compound, which we called LD2, its chemical structure was shown in Fig. 3A. We found that LD2 strongly inhibited GRK3’s kinase activity with the half-maximal inhibitory concentration (IC50) of 82 nM (Fig. 2B). LD2’s inhibition of GRK3 with IC50 of 82 nM was much stronger than inhibition of GRK2 with IC50 of 1954 nM (Fig. 2C), indicating the specificity of LD2 on GRK3. The specificity of LD2 targeting GRK3 was further confirmed when treating MKN45-GRK3 OE cells and control cells using LD2 at different dosage and found that LD2 significantly suppressed tumor cell growth in MKN45-GRK3 OE cells, but no effects in MKN45-GFP control cells (Supplementary Fig. 4A). Interestingly, we found that LD2 significantly suppressed tumor cell growth, colony formation,, and invasion,in a dose-dependent manner in patients-derived GA0518 and GA0804 cells, as well as AGS cell line (Fig. 3D-F, and Supplementary Figs. 4B & 4C). Most importantly, LD2 also suppressed GAC cells invasion induced by overexpression of GRK3 in both KATO III and MKN45 cells (Fig. 3G & 3H), suggesting that GRK3 played a critical role in regulating phenotypic aggressiveness of GAC, while LD2 could curtail the malignant phenotype induced by GRK3.

### GRK3 was positively associated with YAP1 in GAC tissues

**Fig. 4:**
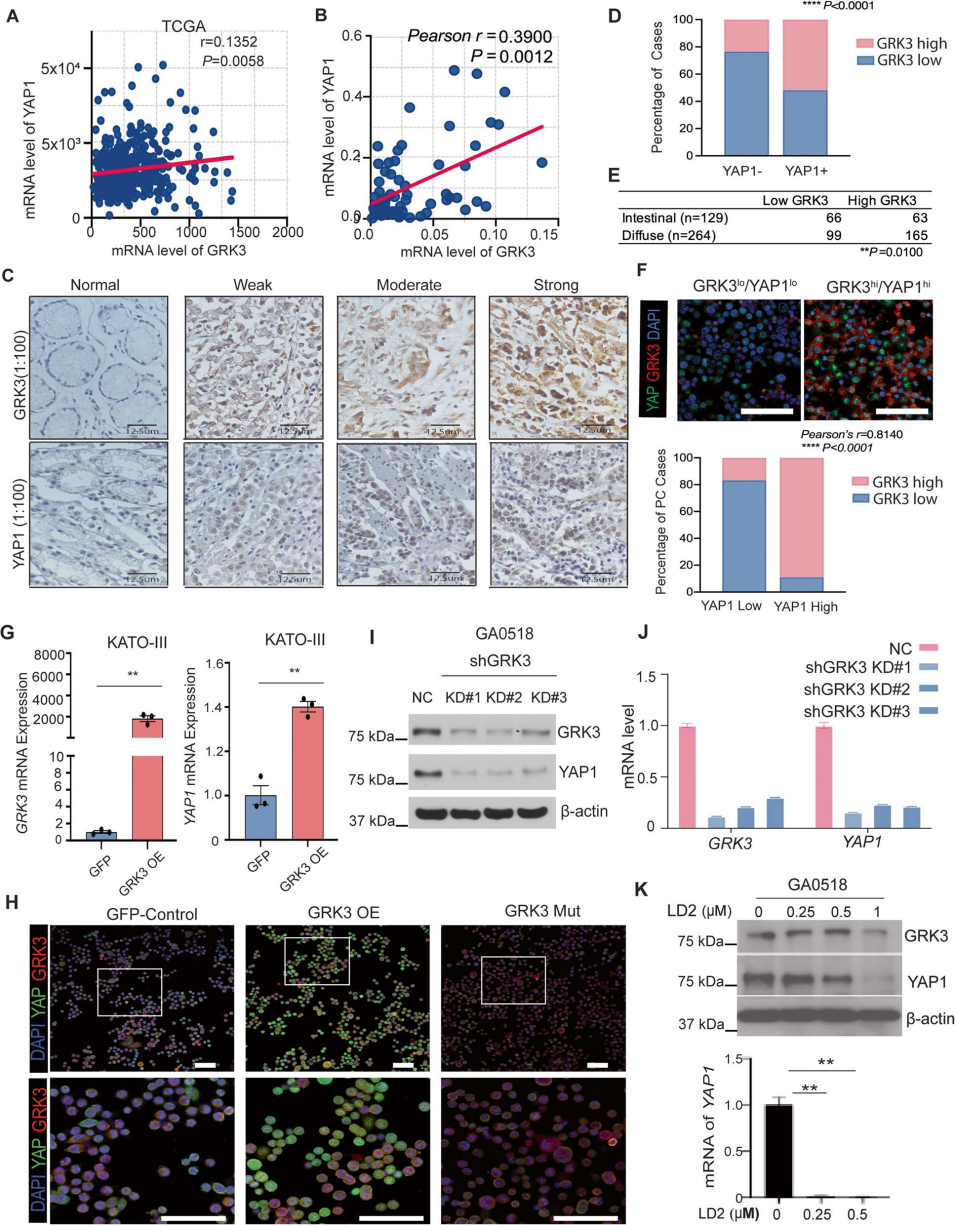
GRK3 and YAP1 expression positively correlated in GAC tissues and GRK3 induced YAP1 expression in GAC cells. **A** The correlation between *GRK3* and *YAP1* in TCGA human STAD patients (*n* = 415). Pearson’s correlation test was used. *P* = 0.0058. **B** qPCR performed in tumor tissues and malignant ascites cells from 66 GAC cases in our cohort showed that GRK3 positively correlated with YAP1 in GAC and peritoneal metastasis. *r* = 0.5082, *P* < 0.001. **C** Representative images on IHC staining of YAP1 and GRK3 proteins showed that expression of YAP1 and GRK3 was positively associated within tumor tissues. **D** Chi-square test analysis indicated a significant positive association between GRK3 and YAP1 expression in the same cohort of TMAs. **E** The correlation of GRK3 expression with histological type was analyzed in TMAs. *P* < 0.01. **F** GRK3 and YAP1 expression was detected using co-immunofluorescent staining in 49 cases of malignant ascites cells; Representative images and quantification of GRK3^lo^/YAP1^lo^ and GRK3^hi^/YAP1^hi^ are shown. *r* = 0.8140; *P* < 0.0001. Scale bar: 25µm. **G** Overexpression GRK3 in KATO III cells dramatically increased YAP1 expression by qPCR; **P < 0.001; **H** Co-staining of YAP1 and GRK3 in KATO III cells with transfected with either GRK3 OE or mutant GRK3 cDNA compared to control cells; Scale bar:50µm. **I**&**J** Knockdown of GRK3 in GA0518 cells dramatically decreased YAP1 expression as shown by western blot (**I**) and qPCR (**J**) which correspond to the decrease in GRK3 level. **K.** Level of YAP1 and GRK3 was determined using western blot (upper panel) and qPCR (bottom panel) in GA0518 cells treated with LD2 at the dosage indicated. **P < 0.001

As demonstrated by us and others, the Hippo/YAP1 pathway plays an essential role in GAC progression, cancer stem cell (CSC) attributes, and PC formation [17, 18]. To explore the relationship between GRK3 and YAP1 in GAC tumors, first, we found that there was a positive correlation between GRK3 and YAP1 mRNA level in TCGA STAD patients (Fig. 4A). Then, we measured the expression of GRK3 and YAP1 in 66 cases of GAC tissues and PC specimens using qPCR. Pearson correlation analysis showed that the expression of GRK3 was significantly associated with YAP1 expression (*r* = 0.3900, *P* = 0.0012; Fig. 4B). This correlation between GRK3 and YAP1 was further validated in GAC TMAs using IHC for GRK3 and YAP1 antibodies (Fig. 4C). Chi-square test analysis further indicated a strong positive association between GRK3 and YAP1 expression (Fig. 4D and Supplementary Table 5). Further analyses of GRK3 staining on GAC TMA revealed that GRK3 was significantly associated with diffuse type of GAC which is more aggressive than intestinal subtype (Fig. 4E). Moreover, we have dual-stained YAP1 and GRK3 in 49 PC metastatic cases and found that both GRK3 and YAP1 were positively correlated (Fig. 4F; *P* < 0.0001). Further, overexpression of GRK3 in KATO III cells increased YAP1 transcription and protein expression (Fig. 4G and J), while KD of GRK3 in GA0518 cells dramatically decreased YAP1 expression and transcription in 3 individual GRK3-KD clones by Western blotting and qPCR respectively, and these decreases corresponded to the decreased level of GRK3 (Fig. 4H and I). Additionally, we have performed rescue experiment in KATO III and MKN45 GRK3 OE cells by knock down YAP1 using LentiCRISPR/CAS9 and found that GRK3 OE significantly increased tumor cell invasion and tumor sphere formation in both KATO III and MKN45 cells, while KD YAP1 in GRK3 OE cells dramatically reduced the capability of invasion and tumor sphere formation induced by GRK3 OR in both KATO III and MKN45 cell lines (Supplementary Figs. 5A-5D). Moreover, pharmacologic suppression of GRK3 using LD2 dramatically inhibited YAP1 protein expression and transcription in GA0518 cells (Fig. 4K). IF staining of YAP1 and GRK3 in GA0518 cells showed that expression of both YAP1 and GRK3 was dramatically decreased in cells treated with LD2 compared to that control cells (Supplementary Fig. 6A). In addition, LD2 significantly decreased YAP1 promoter-luciferase activity when GA0518 cells were transfected with YAP1 promoter-luciferase construct and then treated with LD2, which is in line with the reduction of YAP1 mRNA by LD2 (Supplementary Fig. 6B). Of note, LD2 also significantly inhibited YAP1/Tead transcriptional activity when GA0518 cells were co-transfected with Tead binding elements-luciferase construct and YAP1 cDNA, followed by LD2 treatment (Supplementary Fig. 6C). These results indicated that LD2 suppressed YAP1’s own transcription and its downstream transcriptional activity.

### GRK3 regulated YAP1 targets and inhibition of GRK3 reduced CSC attributes and aggressive phenotype of GAC cells

**Fig. 5:**
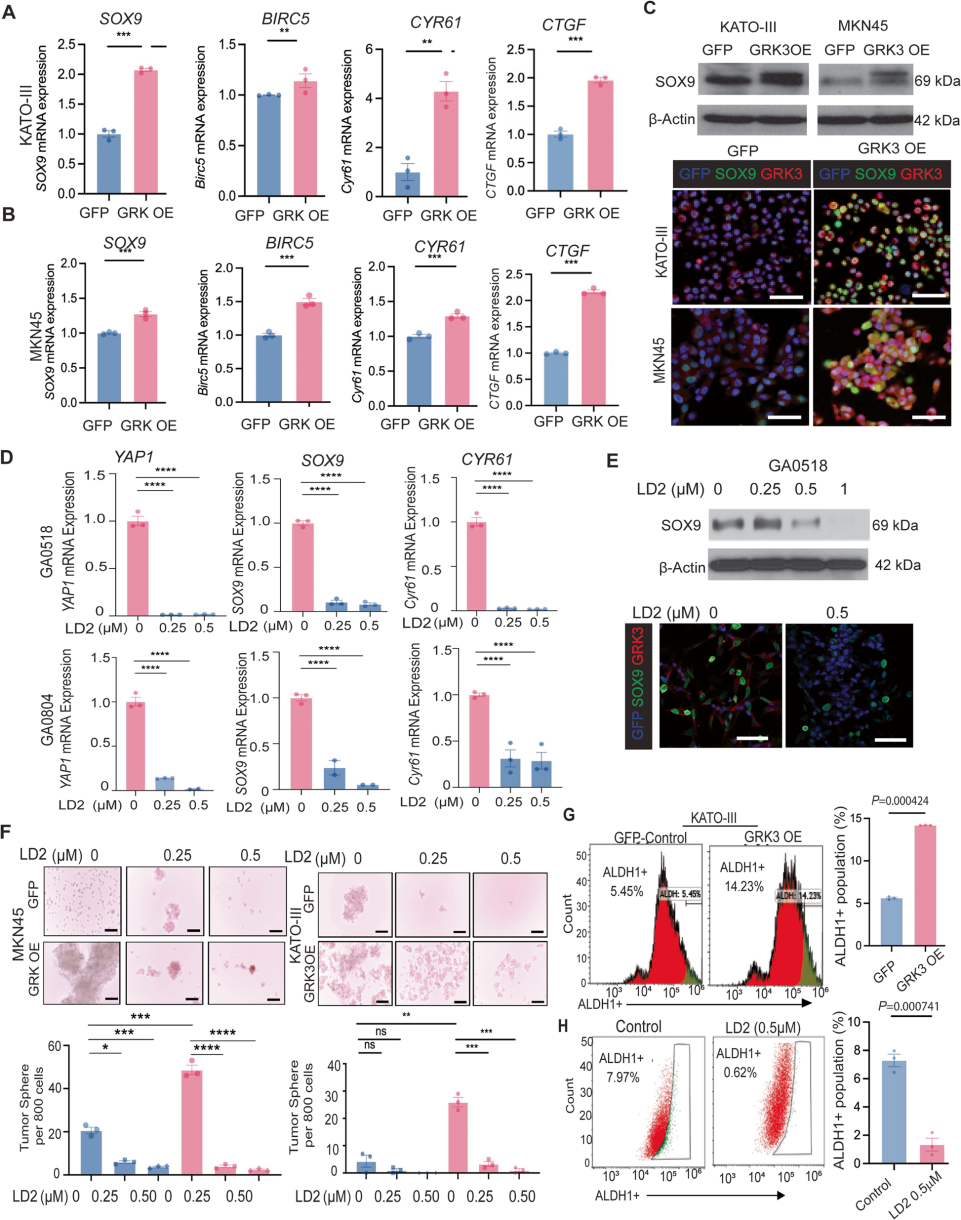
GRK3 positively regulated YAP1 targets and GRK3 inhibitor reduced CSC attributes and aggressive phenotypes of GAC cells. **A** *SOX9*, *Birc5*, *Cyr61* and *CTGF* were detected by qPCR in KATO III cells with or without GRK3 OE; **P < 0.001. **B** *SOX9, Birc5, Cyr61* and *CTGF* were detected by qPCR in MKN45 cells with or without GRK3 OE; ***P < 0.0001.**C** SOX9 expression was detected by Western blot (upper panel) or co-IF in both KATO III and MKN45 cells with or without GRK3 cDNA overexpression (Bottom panel). Scale bar: 50µm. **D** The level of YAP1 and its targets *SOX9, Cyr61* were detected using qPCR in GA0518 and GA0804 patient derived cells treated with or without LD2 at dosage indicated. ****P < 0.0001. **E** Level of SOX9 was determined by Western blot (upper panel) and co-IF (bottom panel) in GA0518 cells and treated with LD2 at indicated dosage. Scale bar: 50µm. **F** Tumor sphere formation was detected in MKN45 and KATO III cells with or without overexpression of GRK3 and then treated with LD2 at indicated dosage. *P < 0.01, **P < 0.001, ***P < 0.0001. **G** Representative image (left) and quantification (right) of ALDH1A1^+^ population was labeled using the ALDEFLUOR kit in KATO III cells with GRK3 OE compared to the control (*n* = 3 biological replicates). *P* = 0.000424. **H** Representative image (left) and quantification (right) of ALDH1A1^+^ population was labeled using the ALDEFLUOR kit in GA0518 cells with LD2 treatment at 0.5µM for 48 h (*n* = 3 biological replicates). *P* = 0.000741

Previously, we and others have reported that Hippo/YAP1 mediates CSC attributes and tumor progression through regulation of its downstream targets, such as SOX9, Birc5, and Cyr61 [17, 23]. We postulated that GRK3 alters CSC attributes and GAC progression through YAP1-mediated downstream signaling. We first determined whether YAP1 down-stream targets, including SOX9, Birc5, Cyr61 and CTGF, were affected by GRK3 genetic alteration. As shown in Fig 5A and B, YAP1 targets, SOX9, Birc5, Cyr61 and CTGF, were significantly upregulated upon overexpression of GRK3 in both KATO III and MKN45 cells (Fig. 5A and B, Supplementary Figs. 7A,7B and 7C). We further showed that SOX9 protein level was dramatically increased in both KATO III and MKN45 with overexpression of GRK3 (Fig. 5C), suggesting that GRK3 positively regulated SOX9, an important stem cell factor [24, 25]. Intriguingly, pharmacological inhibition of GRK3 by LD2 significantly decreased YAP1 and its targets SOX9 and Cyr61 in a dose dependent manner in both GA0518 and GA0804 patient-derived PC cells (Fig. 5D and E). To explore whether GRK3 inhibition affected the function of CSCs, tumor sphere formation was examined in 3 GAC cell lines with high GRK3 and YAP1 expression after treatment with LD2 at various concentrations. Tumor sphere formation in GA0518, GA0804 and AGS was significantly reduced by LD2 treatment in a dose-dependent manner (Supplementary Fig. 8A). Of note, GRK3 overexpression in MKN45 and KATO III significantly increased tumor sphere formation in both GAC cells, which were dramatically suppressed by LD2 treatment in a dose dependent manner (Fig. 5F). ALDH1, a reliable CSC marker in tumor cells, reflects the CSC-like characteristics of various cancer types [26]. To examine the direct association of GRK3 with CSCs traits, we performed ALDH1 labeling in KATO III cells with or without GRK3 OE and found that ALDH1^+^ population is significantly increased in GRK3 OE cells compared to GFP-control cells while inhibition of GRK3 by LD2 dramatically decreased ALDH1^+^ population in three cell lines (Fig. 5G, 5H and Supplementary Fig. 8B).

### GRK3 promoted tumor growth, while inhibition of GRK3 by LD2 significantly suppressed growth in the PDX model

**Fig. 6:**
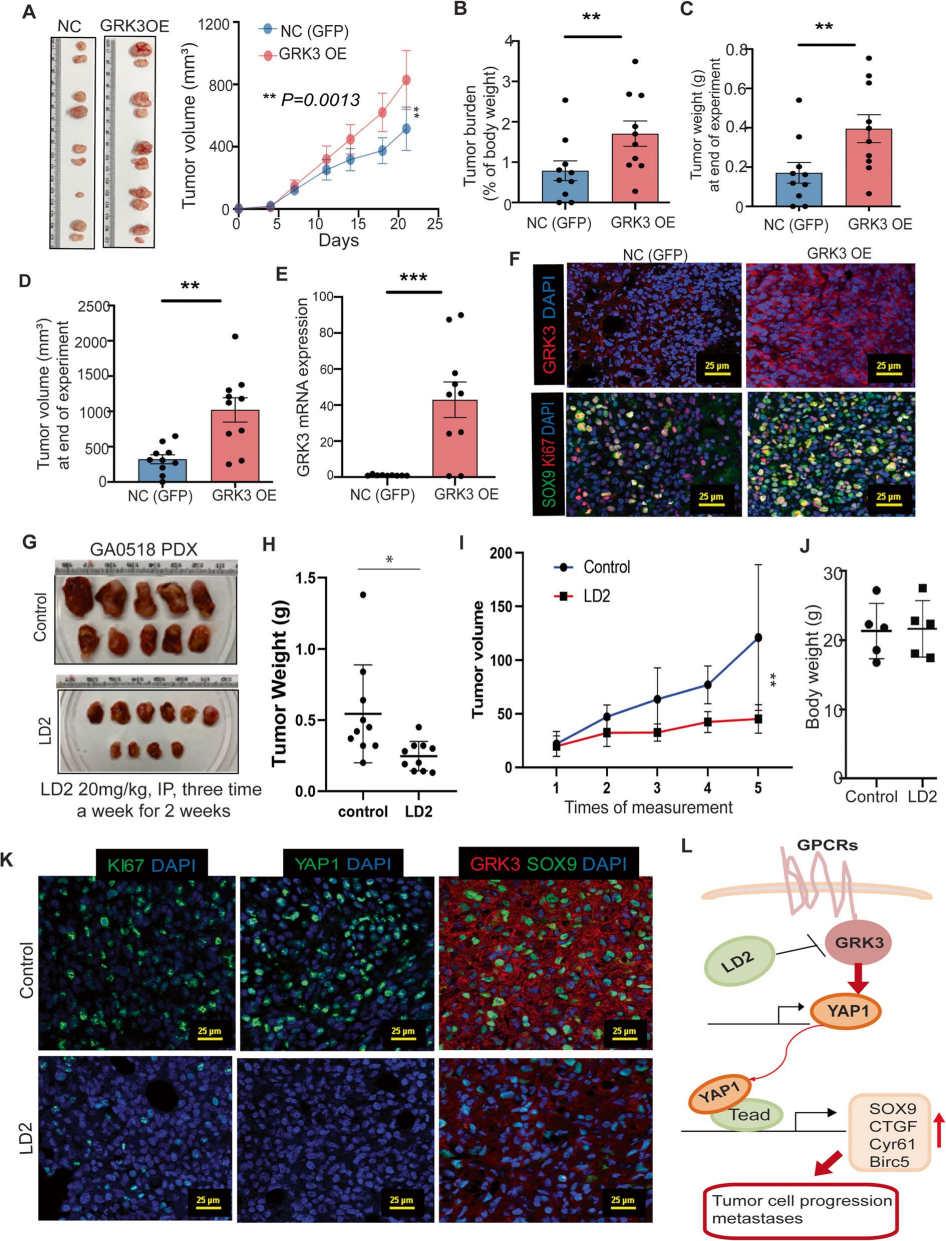
GRK3 promoted tumor growth, while inhibition of GRK3 by LD2 significantly suppressed GAC growth in xenografts. **A** Representative tumor images and tumor volume were shown in MKN45 GRK3 overexpression (OE) vs negative control group (NC) over three-week period after implantation. **B**-**D** Tumor burden, tumor weights and tumor volumes were shown at end point. **E** GRK3 level was detected by qPCR in treated tumor tissues compared to NC group. ***P < 0.001. **F** Co-immunofluorescent staining of GRK3 and SOX9 was performed in tumors of GRK3 OE group vs NC group. Representative images were shown. Scale bar: 25µm. **G** Representative GA0518 xenograft tumors in LD2 treatment group vs control were shown after 2-week treatment. **H** & **I** tumor weights (**H**) and tumor volumes (**I**) were significantly reduced in the LD2-treated group compared with that of the control group. *P < 0.01; **P < 0.001. **J** Mouse body weight was measured in the indicated group. **K** Immunofluorescent staining of GRK3, YAP1, SOX9 and proliferation marker Ki67 was performed in LD2 treated tumor tissues compared with control tumor tissues. Images were captured by confocal microscopy. Scale bar: 25µm. **L** Diagram demonstrates the link between GRK3 and YAP1 and its downstream targets. Novel GRK3 inhibitor LD2 can curtail tumor cell progression and metastases mediated by the GRK3-YAP1 axis

To investigate the impact of GRK3 in GAC *in vivo*, we first determine the effects of overexpression of GRK3 in GAC cells in tumorigenicity. SCID mice were subcutaneously injected with MKN45 cells overexpressing GRK3 or GFP control (NC), which have been validated using qPCR and western blot (Fig. 2B). Over the three-week of inoculation period, tumor growth was faster in GRK3 OE  group compared to NC group. Tumor burden, tumor volume, and tumor weight at end of experiments were also significantly larger in GRK3 OE group than NC group (Fig. 6A-D). Correspondingly, *Grk3* mRNA level was significantly increased in GRK3 OE tumors compared to NC tumors (Fig. 6E). Co-immunofluorescent staining (IF) further validated that expression of GRK3 and SOX9 was dramatically increased in tumor tissues from the GRK3 OE group, compared to the NC group (Fig. 6F). To evaluate the anti-tumor activity of LD2 *in vivo*, we used GA0518 patient-derived PC cells which have high GRK3 and YAP1 expression. SCID mice implanted with GA0518 were randomly assigned to receive either control or LD2 treatment (20 mg/kg, daily, 5 times/week for 2 weeks by intraperitoneal injection). Tumor volumes were observed and measured throughout treatment, and tumor weights and mouse body weights were measured at the end of treatment. The tumor volumes and tumor weights of the LD2-treated group showed a significant reduction compared with those of the control group (Fig. 6G-I). In contrast, the mouse body weights did not differ significantly between the 2 groups (Fig. 6J), indicating that LD2 may be a relatively safe drug. Furthermore, we evaluated expression of GRK3, YAP1, and SOX9 and cell proliferation marker Ki67 in the tumor tissues of mice treated with or without LD2 using IF. All these markers were significantly reduced by LD2 treatment (Fig. 6K). These results confirmed that GRK3 inhibition by LD2 efficiently suppressed GAC tumor growth *in vivo.*

To further demonstrate the effect of GRK3 in the PC-related mouse metastatic model (the tumor microenvironment is somewhat similar to that of metastatic human GAC), we injected MKN45 GFP control (NC) cells and GRK3 OE cells intraperitoneally in SCID mice. Our data showed that GRK3 OE in MKN45 cells significantly promoted PC measured by bioluminescence weekly, while LD2 treatment significantly reversed PC in GRK3 OE group (Supplemental Fig. 9) indicating that CRK3 is critical for peritoneal metastases and LD2 suppressed PC induced by GRK3 OE.

## Discussion

Critical knowledge in new regulators and therapeutic targets for GAC patients is urgently needed. Through shRNA and cDNA functional screening of human kinases, we previously discovered that GRK3 is an essential kinase for prostate cancer progression. We found that GRK3 is significantly higher in GAC tumor tissues verse normal from TCGA dataset indicating its potential role in GAC. However, its expression and functions in GAC progression and metastases remain unclear. In the present study, for the first time, we showed that GRK3 expression was upregulated in GAC tissues and that higher GRK3 expression was significantly associated with higher TNM stage, more lymph node metastases, diffuse phenotype, and shorter survival. Importantly, we identified a novel GRK3 inhibitor LD2, which demonstrated strong anti-tumor activity *in vitro* and *in vivo* in the PDX model. Inhibition of GRK3, either pharmacologically or by genetic KD, in patient-derived PC cells resulted in decreased cell proliferation, invasion, colony formation, and CSC attributes, while overexpression of GRK3 did the opposite. All these results suggest that GRK3 plays an oncogenic role in GAC. Moreover, we discovered a positive association between GRK3 and YAP1, and GRK3 upregulation in GAC cells increased YAP1 and its downstream targets, while GRK3 inhibition led to decreased in YAP1 and its downstream targets (Fig. 6L). Altogether, our study indicates that GRK3 is a poor prognosticator and a novel therapeutic target against GAC. Targeting GRK3 with LD2 may diminish aggressive phenotypes of GAC.

The clinical significance of GRK3 expression in different tumor types varies drastically and seems context-dependent. To our knowledge, our study is the first to report GRK3 expression in a large cohort of GAC patients, consisting of 393 pairs of the primary tumor and non-tumor adjacent tissues, as well as PC cells from metastatic GAC patients. By IHC and immunofluorescent staining, we found that GRK3 expression was higher in tumor tissues than in adjacent normal tissues and even higher in PC cells. Further analysis revealed the overexpression of GRK3 in GACs of patients with diffuse type, lymph node metastases, and those with higher tumor stage. These results are consistent with findings reported in colon cancer and prostate cancer [7, 12], although in breast cancer, decreased GRK3 expression correlates with worse phenotype and liver metastases [27]. Overall, our functional studies using pharmacologic and genetic modulations of GRK3 revealed that GRK3 plays an important oncogenic role in GAC, as the inhibition of GRK3 reduced GAC's aggressive phenotypes, including cell proliferation, tumor sphere formation, and invasion.

Mounting evidence suggests that GPCR regulates cell proliferation, stemness, self-renewal, and survival by modulating core cascades related to these processes, including small GTPases, MAPK, and PI3K/AKT/mTOR pathways, as well as Wnt/β-catenin or YAP/TAZ-dependent transcription programs [28–31]. Seven GPCR kinases (GRKs) in human genome critically regulate signaling pathways and functions of nearly 1,000 GPCRs [28, 32]. GRK3 is one of the 7 ubiquitously expressed GRKs that regulate the functionality of both G protein-coupled receptors (GPCR) and growth factor receptors and to directly control cytosolic, cytoskeletal or nuclear signaling components of pathways relevant for these processes [28]. GRK3 has been reported to phosphorylate the agonist-occupied form of β-adrenergic receptors and several other GPCRs to regulate their signaling either positively or negatively [33–36]. GRK3 may promote cancer development by facilitating GPCR-mediated oncogenic functions through phosphorylation of some GPCRs.

To investigate mechanisms by which GRK3 exerts its oncogenic functions in GAC, we focused on GRK3-YAP1 axis, due to the importance of Hippo/YAP1 signaling in GAC, such as conferring CSC attributes and promoting metastases. Several lines of evidence from our study support this postulation. First, there was a strong positive correlation between GRK3 and YAP1 upon IHC staining in TMAs, which was confirmed using qPCR in a separate patient cohort as well as TCGA dataset, including primary tumors and PC cells. Second, genetic or pharmacologic inhibition of GRK3 dramatically decreased YAP1 expression and its targets SOX9, Birc5, CYR61, and CTGF, accompanied by a reduction in aggressive phenotypes and CSC attributes in GAC cell lines and patient-derived cells. Conversely, GRK3 overexpression in both KATO III and MKN45 induced YAP1 targets and increased aggressive phenotypes of GAC cells. More detailed mechanisms on how GRK3 regulates the activation of YAP1 signaling remains a subject of active investigation in our laboratories.

As kinases have proven to be suitable drug targets for cancer therapeutics [34], to translate our findings into the clinic, it is necessary to identify an effective GRK3 kinase inhibitor. By collaborating with Dr. Kevin Dalby’s laboratory at The University of Texas at Austin, we screened a compound library to identify GRK3 kinase inhibitors using recombinant GRK3 protein and Z-LYTE *in vitro* kinase assay. We identified LD2, which potently inhibited GRK3 kinase activity (Figs. 3B & 3C). Of note, GRK2, the GRK3’s closest sibling kinase, is much less potently inhibited by LD2 than GRK3. Interestingly, the structure of LD2 (Fig. 3A) coincides with that of PF-477736, a CHK1 inhibitor [35, 36]. Importantly, we found that LD2 suppresses GRK3-mediated oncogenic activity in GAC cell lines and patient PC cells, whose inhibitory effects are less dramatic in GRK3-low cancer cells. LD2, a novel GRK3 inhibitor, actively reduced tumor cell growth, invasion, and tumor sphere formation *in vitro* in multiple GAC cell models and *in vivo* in a GAC PDX model with high GRK3 and YAP1 expression. The evidences here provide a strong rationale for targeting GRK3 with LD2 and its derived compounds in GAC patients. Details on the mechanism action of LD2 and the relationship between inhibitions of CHK1 and GRK3 kinase activities warrant further investigation.

## Conclusion

In conclusion, we uncovered, for the first time to our knowledge, that GRK3 is over-expressed in primary and metastatic GAC tumors compared with adjacent normal tissues, where its overexpression is significantly associated with poor prognosis and shorter survival. Functional studies using genetic and pharmacologic approaches in the various GAC cell models and patient-derived cells confirm that GRK3 plays an oncogenic role. Furthermore, we have discovered a GRK3 inhibitor, LD2, that suppresses GAC aggressive attributes and tumor growth *in vitro* and *in vivo.* Thus, GRK3 could serve as a prognostic biomarker and a therapeutic target for advanced GAC patients who are in desperate need of better treatments.

## Supplementary Information

**Additional file 1: Supplementary Table 1.** Designed guide RNA for GRK3 or YAP1knock-down. **Supplementary Table 2.** Primer sequences used in this study. **Supplementary Table 3.** Expression of GRK3 with patients’ characteristics. **Supplementary Table 4.** Univariate and Multivariate analysis of GRK3 in GAC patients. **Supplementary Table 5.** The correlation between GRK3 and YAP1 expression in GAC. 

**Addiitional file 2: Supplementary Figure1.** GRK3 expression in PDX tumors of GAC and paired primary tumor and PC. A. Diagram demonstrates the several patients’ PC cells (malignant ascites cells) into nude mice to generate PDXs (left). Western blot of GRK3 expression of Primary and ascites from PDX tumor generated from GAC patient (right). B. Representative co-immunofluorescent staining images of GRK3 and SOX9 expression on more PC specimen. Scale bars, 25µm. C. Representative co-immunofluorescent staining images of GRK3 and SOX9 expression on paired human primary tumor and PC samples. Scale bars, 25µm.  **Supplementary Figure 2.** Overexpression of GRK3 in GAC cells increased malignant behaviors of GAC. A. Cell viability assay (MTS) was performed in MKN45-GRK3 and MKN45-GFP control cells. Fold increases of cell growth in day 6 and day 8 in media with the indicated FBS % was plotted on y-axis. **P<0.001.  B. Tumor sphere formation assay was performed in MKN45 GFP and GRK3 OE cells (*n* = 3 biological replicates). *P<0.01. C. Invasion assay was performed in KATO III GFP, GRK3 OE and GRK3 Mut cells (*n* = 3 biological replicates) as described in Materias&Methods. *P<0.01; **P<0.001. Scale bar: 25µm. D. Colony formation assay was performed in KATO III GFP, GRK3 OE and GRK3 Mut cells (*n* = 3 biological replicates) as described in Materias&Methods. Scale bar: 100µm. **P<0.01; ***P<0.001. E. GFP and GRK3 OE cells of KATO III was cultured in 96-well plate, and MTS assay was performed at Day 3, 5, 7 and 9. **P<0.01; ***P<0.001. F. Tumor sphere assay was performed in NC and GRK KD cells of GA0518. G. AGS cells seeded in a 96-well plate were infected with a series of shRNA of GRK3 and scramble virus doses, from 20ul to 2.5ul per well. Cell survival and proliferation were measured by alamar blue viability assay 7 days after infection. X-axis represents the series of shRNA virus doses in the infections. Values on Y-axis are relative growth which was normalized to the scramble control shRNA for each virus dose for either cell line. *P* values were calculated using 2-sided Student’s t-test. ***: *P* < 0.005; *: *P* < 0.05. **Supplementary Figure 3.** Flow charts of our compound library screening for identifying GRK3 inhibitors A. Schematic illustration of our compound library screening to identify candidates of GRK3 inhibitors. B. Detailed flow chart of the library screening, using Z'-LYTE Kinase Assay (the Ser/Thr 16 Peptide Kit from Life Technology). GRK3 recombinant protein was first mixed with diluted compound library, followed by the addition of ATP, peptide substrate and Z-LYTE buffer. Effective GRK3 inhibitors were identified when they blocked GRK3-mediated phosphorylation of the peptide substrates. Unphosphorylated peptides were cleaved after the addition of Developing Reagent, which resulted in no FRET (Fluorescence Resonance Energy Transfer). **Supplementary Figure 4.** LD2 specifically inhibited the proliferation of GRK3 OE cells in MKN45 and decreased colony formation and invasion in AGS cells. A. Cell viability assay was performed in MKN45 GFP and GRK3 OE cells treated with indicated concertation of LD2. *P<0.01; **P<0.001. B. Colony formation assay was performed in AGS treated with indicated concertation of LD2. *P<0.01; **P<0.001. C. Invasion assay was performed in AGS treated with indicated concertation of LD2. **P<0.001. **Supplementary Figure 5.** KD YAP1 in GRK3 OE GAC cells rescue the malignant phenotype induced by GRK3 OE in both KATO III and MKN45 cells. A&B. Reduced YAP1 in GRK3 OE cells suppress tumor cell invasion induced by GRK3 OE in KATO III and MKN45 cells. C&D. Knockdown YAP1 in GRK3 OE cells suppress tumor sphere formation induced by GRK3 OE in KATO III and MKN45 cells. **Supplementary Figure 6.** LD2 inhibited YAP expression and YAP/TEAD transcriptional activity. A. Representative co-immunofluorescent staining images of GRK3 and YAP expression on GA0518 cell treated with or without LD2 at 0.5µM for 24 hours. Scale bars, 25µm. B. YAP1 promoter activity was determined at 48 hours after co-transfection of YAP1-luciferase promoter and Renilla luciferase and then treated with LD2 at indicated dosage (*n* = 3 biological replicates). **P<0.001. C. YAP1/TEAD transcriptional activity was detected by cotransfection of Gal4-Tead and 5XUAS-luciferase plasmids with YAP1 cDNA into GA0518 cells and then treated with LD2 at indicated dosage. Luciferase reporter activity was measured after 48 hours. (*n* = 3 biological replicates).  (** *P* < 0.01). **Supplementary Figure 7.** Increased expression of YAP1 targets-CTGF, Cyr61 and Survivin (Birc5) in GRK3 OE cells compared to normal control cells (NC) in both MKN45 and KATOIII cells. A. CTGF and GRK3 expression in NC and GRK3 OE cells in both MKN45 and KATO III cell line, examined by immunofluorescence. B. Survivin expression in NC and GRK3 OE cells in both MKN45 and KATO III cell line, examined by immunofluorescence. C. Cyr61 expression in NC and GRK3 OE cells in both MKN45 and KATO III cell line, examined by immunofluorescence. Scale bar: 50 µm. **Supplementary Figure 8.** GRK3 inhibitor LD2 inhibited stem cell trait of GAC. A. Tumor sphere formation assay was performed in GA0518, GA0804 and AGS cells treated with indicated concentration of LD2 (*n* = 3 biological replicates). *P<0.01, **P<0.001, ***P<0.0001. Scale bar 100µm. B. ALDH1A1+ population was labeled using the ALDEFLUOR kit in GA0804 and AGS cells as described in Materials& Methods. **Supplementary Figure 9.** Effects of GRK3 OE on peritoneal metastases, while LD2 decrease peritoneal metastases induced by GRK3 OE in PC metastatic model. A. Peritoneal metastasis were shown in the indicated group. B&C. Representative bioluminescence images and quantification of the mice in the indicated group. *P*=0.045; *P*=0.0079.

## Data Availability

The datasets used and/or analyzed, and materials used during the current study are available from the corresponding author on reasonable request.

## Abbreviations

GPCR: G protein-coupled receptor
GRK3: G protein-coupled receptor kinase 3
GAC: Gastric adenocarcinoma
PC: Peritoneal carcinomatoses (implants or malignant ascites)
TMAs: Tumor tissue microarrays
CMU: China Medical University
MDACC: MD Anderson Cancer Center
qPCR: Quantitative real-time polymerase chain reaction
OE: Overexpression
KD: Knockdown
NC: Control cells
SCID: Severe combined immunodeficient
IHC: Immunohistochemical staining
CSCs: Cancer stem cells

## References

[1] Bray F, Ferlay J, Soerjomataram I (2018). Global cancer statistics 2018: GLOBOCAN estimates of incidence and mortality worldwide for 36 cancers in 185 countries. CA Cancer J Clin.

[2] Wadhwa R, Elimova E, Shiozaki H (2014). Anti-angiogenic agent ramucirumab: meaningful or marginal?. Expert Rev Anticancer Ther.

[3] Ajani JA (2007). Recent developments in cytotoxic therapy for advanced gastric or gastroesophageal carcinoma: the Phase III trials. Gastrointest Cancer Res.

[4] Ajani JA, Lee J, Sano T (2017). Gastric adenocarcinoma. Nat Rev Dis Primers.

[5] Huang KK, Ramnarayanan K, Zhu F (2018). Genomic and epigenomic profiling of high-risk intestinal metaplasia reveals molecular determinants of progression to gastric cancer. Cancer Cell.

[6] Wang K, Yuen ST, Xu J (2014). Whole-genome sequencing and comprehensive molecular profiling identify new driver mutations in gastric cancer. Nat Genet.

[7] Li W, Ai N, Wang S (2014). GRK3 is essential for metastatic cells and promotes prostate tumor progression. Proc Natl Acad Sci U S A.

[8] O'Hayre M, Degese MS, Gutkind JS (2014). Novel insights into G protein and G protein-coupled receptor signaling in cancer. Curr Opin Cell Biol.

[9] Woerner BM, Luo J, Brown KR (2012). Suppression of G-protein-coupled receptor kinase 3 expression is a feature of classical GBM that is required for maximal growth. Mol Cancer Res.

[10] Liu WJ, Zhou L, Liang ZY (2018). High expression of GRK3 is associated with favorable prognosis in pancreatic ductal adenocarcinoma. Pathol Res Pract.

[11] Jin Y, Liang ZY, Zhou WX (2017). Expression and significances of G-Protein-coupled receptor kinase 3 in hepatocellular carcinoma. J Cancer.

[12] Jiang T, Yang C, Ma L (2017). Overexpression of GRK3, promoting tumor proliferation, is predictive of poor prognosis in colon cancer. Dis Markers.

[13] Ajani JA, Xu Y, Huo L (2021). YAP1 mediates gastric adenocarcinoma peritoneal metastases that are attenuated by YAP1 inhibition. Gut.

[14] Song S, Wang Z, Li Y (2020). PPARdelta interacts with the Hippo coactivator YAP1 to promote SOX9 expression and gastric cancer progression. Mol Cancer Res.

[15] Wang RY, Chen XW, Zhang WW (2020). CYP2E1 changes the biological function of gastric cancer cells via the PI3K/Akt/mTOR signaling pathway. Mol Med Rep.

[16] Akhtar M, Cheng Y, Magno RM (2001). Promoter methylation regulates Helicobacter pylori-stimulated cyclooxygenase-2 expression in gastric epithelial cells. Cancer Res.

[17] Song S, Ajani JA, Honjo S (2014). Hippo coactivator YAP1 upregulates SOX9 and endows esophageal cancer cells with stem-like properties. Cancer Res.

[18] Song S, Honjo S, Jin J (2015). The Hippo Coactivator YAP1 Mediates EGFR Overexpression and Confers Chemoresistance in Esophageal Cancer. Clin Cancer Res.

[19] Dong X, Song S, Li Y, et al. Loss of ARID1A activates mTOR signaling and SOX9 in gastric adenocarcinoma-rationale for targeting ARID1A deficiency. Gut. 2021;71:467-478. 10.1136/gutjnl-2020-322660 PMC9724309 33785559

[20] Song S, Ajani JA, Honjo S, et al. Hippo coactivator YAP1 upregulates SOX9 and endows stem-like properties to esophageal cancer cells. Cancer Res. 2014;74:4170-4182. 10.1158/0008-5472.CAN-13-3569 PMC4136429 24906622

[21] Wu P, Nielsen TE, Clausen MH (2015). FDA-approved small-molecule kinase inhibitors. Trends Pharmacol Sci.

[22] Roskoski R (2019). Properties of FDA-approved small molecule protein kinase inhibitors. Pharmacol Res.

[23] Song S, Xie M, Scott AW (2018). A novel YAP1 inhibitor targets CSC-Enriched Radiation-Resistant Cells and Exerts Strong antitumor activity in esophageal adenocarcinoma. Mol Cancer Ther.

[24] Christin JR, Wang C, Chung CY (2020). Stem cell determinant SOX9 promotes lineage plasticity and progression in basal-like breast cancer. Cell Rep.

[25] Song S, Chen Q, Li Y (2021). Targeting cancer stem cells with a pan-BCL-2 inhibitor in preclinical and clinical settings in patients with gastroesophageal carcinoma. Gut.

[26] Christophe Ginestier1 MHH, Emmanuelle Charafe-Jauffret3, Florence Monville3, Julie Dutcher1, Marty Brown1, Jocelyne Jacquemier3, Patrice Viens3, Celina Kleer1, Suling Liu1, Anne Schott1, Dan Hayes1, Daniel Birnbaum3, Max S. Wicha1, and Gabriela Dontu1,. ALDH1 is a marker of normal and malignant human mammary stem cells and a predictor of poor clinical outcome. Cell Stem Cell 2007; 15:555–567. 10.1016/j.stem.2007.08.014 PMC2423808 18371393

[27] Billard MJ, Fitzhugh DJ, Parker JS (2016). G protein coupled receptor kinase 3 regulates breast cancer migration, invasion, and metastasis. PLoS ONE.

[28] Nogues L, Palacios-Garcia J, Reglero C (2018). G protein-coupled receptor kinases (GRKs) in tumorigenesis and cancer progression: GPCR regulators and signaling hubs. Semin Cancer Biol.

[29] Liu Y, An S, Ward R (2016). G protein-coupled receptors as promising cancer targets. Cancer Lett.

[30] Jiang Y, Zhuo X, Mao C. G protein-coupled receptors in cancer stem cells. Curr Pharm Des. 2020;26:1952-1963. 10.2174/1381612826666200305130009 32133959

[31] Bar-Shavit R, Maoz M, Kancharla A, et al. G protein-coupled receptors in cancer. Int J Mol Sci. 2016;17:1320. 10.3390/ijms17081320 PMC5000717 27529230

[32] Yu S, Sun L, Jiao Y (2018). The role of G protein-coupled receptor kinases in cancer. Int J Biol Sci.

[33] Benovic JL, Stone WC, Huebner K (1991). cDNA cloning and chromosomal localization of the human beta-adrenergic receptor kinase. FEBS Lett.

[34] Alhosaini K, Azhar A, Alonazi A (2021). GPCRs: The most promiscuous druggable receptor of the mankind. Saudi Pharm J.

[35] Klaeger S, Heinzlmeir S, Wilhelm M, et al. The target landscape of clinical kinase drugs. Science. 2017;358(6367). 10.1126/science.aan4368 PMC6542668 29191878

[36] Thompson R, Eastman A (2013). The cancer therapeutic potential of Chk1 inhibitors: how mechanistic studies impact on clinical trial design. Br J Clin Pharmacol.

