# Supplementary material for: GRK3 is a poor prognosticator and serves as a therapeutic target in advanced gastric adenocarcinoma
Source: J Exp Clin Cancer Res. 2022 Aug 23;41:257. doi: 10.1186/s13046-022-02463-6 (PMC9396876; doi:10.1186/s13046-022-02463-6)
Supplement: Supplementary file 1 — Additional file 1: Supplementary Table 1. Designed guide RNA for GRK3 or YAP1knock-down. Supplementary Table 2. Primer sequences used in this study. Supplementary Table 3. Expression of GRK3 with patients’ characteristics. Supplementary Table 4. Univariate and Multivariate analysis of GRK3 in GAC patients. Supplementary Table 5. The correlation between GRK3 and YAP1 expression in GAC. [file 13046_2022_2463_MOESM1_ESM.docx]

**Supplementary Table 1.**

Designed guide RNA for GRK3 or YAP1knock-down

| Grk3.gRNA1.F | 5’  caccgGGCCATCAGGTAACTGACAT 3’ |
| --- | --- |
| Grk3.gRNA1.R | 5’ aaacATGTCAGTTACCTGATGGCCc 3’ |
| Grk3.gRNA2.F | 5’  caccgCAGCAAGAGGATCGTCCTGC 3’ |
| Grk3.gRNA2.R | 5’ aaacGCAGGACGATCCTCTTGCTGc 3’ |
| Grk3.gRNA3.F | 5’  caccgGGTACTTCTGCATCACACTC 3’ |
| Grk3.gRNA3.R | 5’ aaacGAGTGTGATGCAGAAGTACCc 3’ |
| YAP1.gRNA1. F | 5’ caccgGCACGATCTGATGCCCGGCG 3’ |
| YAP1.gRNA1. R | 5’ aaacCGCCGGGCATCAGATCGTGCc 3’ |
| YAP1.gRNA2. F | 5’ caccgTGGGGCACGTTGGCCGTCTT 3’ |
| YAP1.gRNA2. R | 5’ aaacAAGACGGCCAACGTGCCCCAc 3’ |
| YAP1.gRNA3. F | 5’ caccgGCCGGTTGCCCGGGTCCGGA 3’ |
| YAP1.gRNA3. R | 5’ aaacTCCGGACCCGGGCAACCGGCc 3’ |

**Supplementary Table 2.**

Primer sequences used in this study

| GRK3 primers: forward | 5’ GCAGTGCCGACTGGTTCT 3’ |
| --- | --- |
| GRK3 primers: reverse | 5' GTCTGAAAGGGCTGTGACCT 3' |
| YAP1 primers: forward | 5’ CTGTCCCAGATGAACGTCAC 3’ |
| YAP1 primers: reverse | 5’ TTCTCTGGTTCATGGCAAAA 3’ |
| SOX9 primers: forward | 5’ GTAATCCGGGTGGTCCTTCT 3’ |
| SOX9 primers: reverse | 5’ GTACCCGCACTTGCACAAC 3’ |
| CTGF primers: forward | 5’ GTGTGCACCGCCAAAGATGG 3’ |
| CTGF primers: reverse | 5’ AGGCACGTGCACTGGTACTT 3’ |
| Cyr61 primers: forward | 5’AACGAGGACTGCAGCAAAA 3’ |
| Cyr61 primers: reverse | 5’ TATTCACAGGGTCTGCCCTC 3’ |
| hGAPDH-5 | 5’ ACCCAGAAGACTGTGGATGG 3’ |
| hGAPDH-3 | 5’ TCTAGACGGCAGGTCAGGTC 3’ |

**Supplementary Table 3. Expression of GRK3 with patients’ characteristics**

| **GRK3** | Frequency | No. of patients (%) | | P |
| --- | --- | --- | --- | --- |
|  |  | -(165) | +(228) |  |
| Age |  |  |  | 0.140 |
| median | 61 |  |  |  |
| Minimum-maximum | 27-85 |  |  |  |
| Sex |  |  |  | 1.000 |
| male | 292 | 126 | 166 |  |
| female | 101 | 39 | 62 |  |
| Size |  |  |  | 0.731 |
| Median(cm) | 5 |  |  |  |
| Minimum-maximum(cm) | 0.3-17 |  |  |  |
| Differentiation |  |  |  | 0.044 |
| well | 13 | 8 | 5 |  |
| moderate | 116 | 57 | 59 |  |
| Poor | 264 | 100 | 164 |  |
| Lauren |  |  |  | 0.010 |
| diffused | 264 | 99 | 165 |  |
| intestinal | 129 | 66 | 63 |  |
| T category |  |  |  | 0.569 |
| T1 | 1 | 0 | 1 |  |
| T2 | 25 | 9 | 16 |  |
| T3 | 67 | 28 | 39 |  |
| T4 | 300 | 128 | 172 |  |
| N category |  |  |  | 0.001 |
| N0 | 76 | 40 | 36 |  |
| N1 | 92 | 46 | 46 |  |
| N2 | 77 | 33 | 44 |  |
| N3 | 148 | 46 | 102 |  |
| M category(M1 vs M0) |  |  |  | 0.127 |
| M0 | 266 | 119 | 147 |  |
| M1 | 127 | 46 | 81 |  |
| TNM stage |  |  |  | 0.043 |
| I | 11 | 4 | 7 |  |
| II | 71 | 38 | 33 |  |
| III | 184 | 77 | 107 |  |
| IV | 127 | 46 | 81 |  |
| Vein invasion |  |  |  | 1.000 |
| + | 390 | 164 | 226 |  |
| - | 3 | 1 | 2 |  |

| **GRK3** | Univariate | | | Multivariate | | |
| --- | --- | --- | --- | --- | --- | --- |
|  | HR | 95%CI | P | HR | 95%CI | P |
| Age(continue) | 1.011 | 0.997-1.025 | 0.140 |  |  |  |
| Sex (male vs. female) | 0.927 | 0.680-1.262 | 0.628 |  |  |  |
| Size(continue) | 1.096 | 1.040-1.155 | 0.001 | 1.035 | 0.977-1.095 | 0.243 |
| Differentiation |  |  | 0.056 |  |  |  |
| well | 1 |  | 0.061 |  |  |  |
| moderate | 1.348 | 0.772-2.323 | 0.410 |  |  |  |
| poor | 1.423 | 0.855-2.368 | 0.035 |  |  |  |
| Lauren(intestinal vs. diffused) | 0.793 | 0.590-1.065 | 0.124 |  |  |  |
| T category |  |  | 0.007 |  |  | 0.801 |
| T1 | 1 |  | 0.029 | 1 |  |  |
| T2 | 0.656 | 0.254-1.692 | 0.869 | 0.331 | 0.036-3.039 | 0.805 |
| T3 | 0.987 | 0.444-2.193 | 0.859 | 0.435 | 0.049-3.878 | 0.872 |
| T4 | 1.391 | 0.655-2.953 | 0.851 | 0.437 | 0.050-3.847 | 0.869 |
| N category |  |  | <0.001 |  |  | <0.001 |
| N0 | 1 |  | <0.001 | 1 |  |  |
| N1 | 1.300 | 0.778-2.173 | 0.317 | 1.182 | 0.702-1.991 | 0.530 |
| N2 | 1.703 | 1.021-2.839 | 0.041 | 1.472 | 0.874-2.481 | 0.344 |
| N3 | 3.857 | 2.478-6.003 | <0.001 | 3.083 | 1.933-4.917 | <0.001 |
| M category(M1 vs. M0) | 1.893 | 1.439-2.490 | <0.001 | 1.601 | 1.205-2.127 | 0.001 |
| Vein invasion(+ vs. -) | 1.479 | 0.367-5.054 | 0.582 |  |  |  |
| GRK3(+ vs. -) | 1.563 | 1.176-2.079 | 0.002 | 1.257 | 0.938-1.683 | 0.126 |

**Supplementary Table 4. Univariate and Multivariate analysis of GRK3 in GAC patients**

**Supplementary Table 5. The correlation between GRK3 and YAP1 expression in GAC**

|  | **GRK3** | | |  |
| --- | --- | --- | --- | --- |
| **YAP1** | Negative | Positive | Total | P<0.001 |
| Negative | 123 | 115 | 238 |  |
| Positive | 43 | 141 | 184 |  |
| Total | 166 | 256 | 422 |  |
