# Supplementary material for: GRK3 is a poor prognosticator and serves as a therapeutic target in advanced gastric adenocarcinoma
Source: J Exp Clin Cancer Res. 2022 Aug 23;41:257. doi: 10.1186/s13046-022-02463-6 (PMC9396876; doi:10.1186/s13046-022-02463-6)

Supplementary Figure 1.

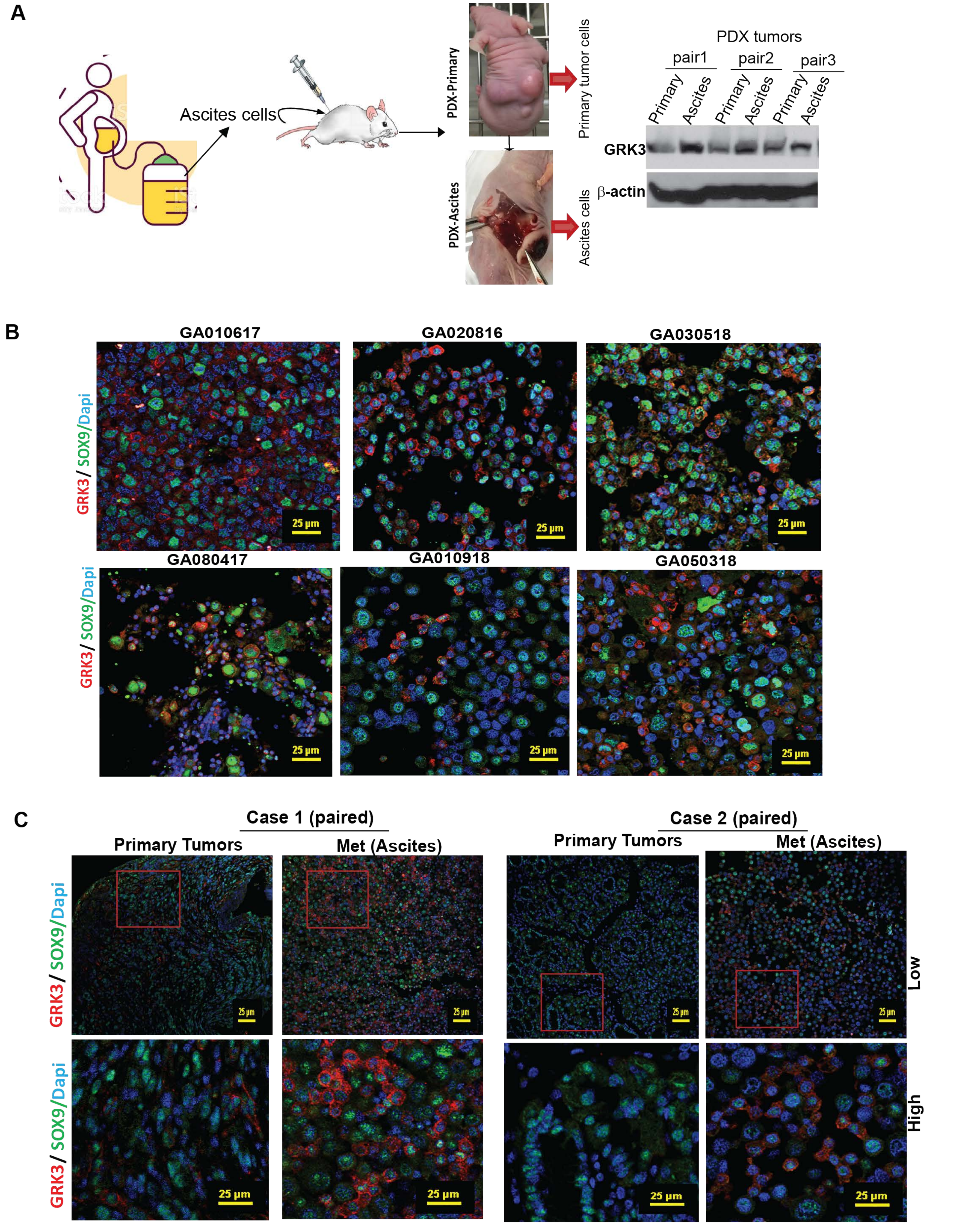

Supplementary Figure 2.

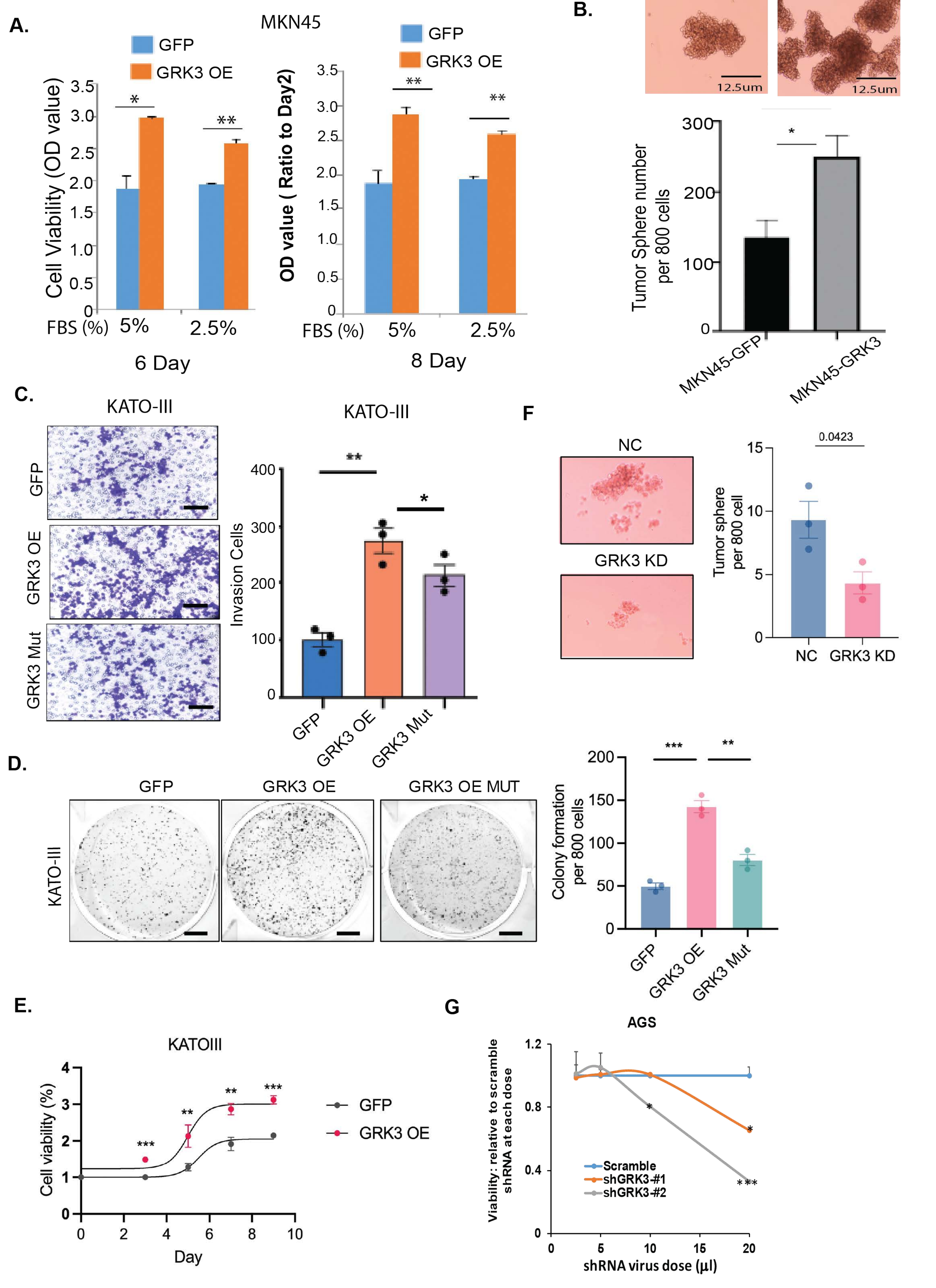

Supplementary Figure 3.

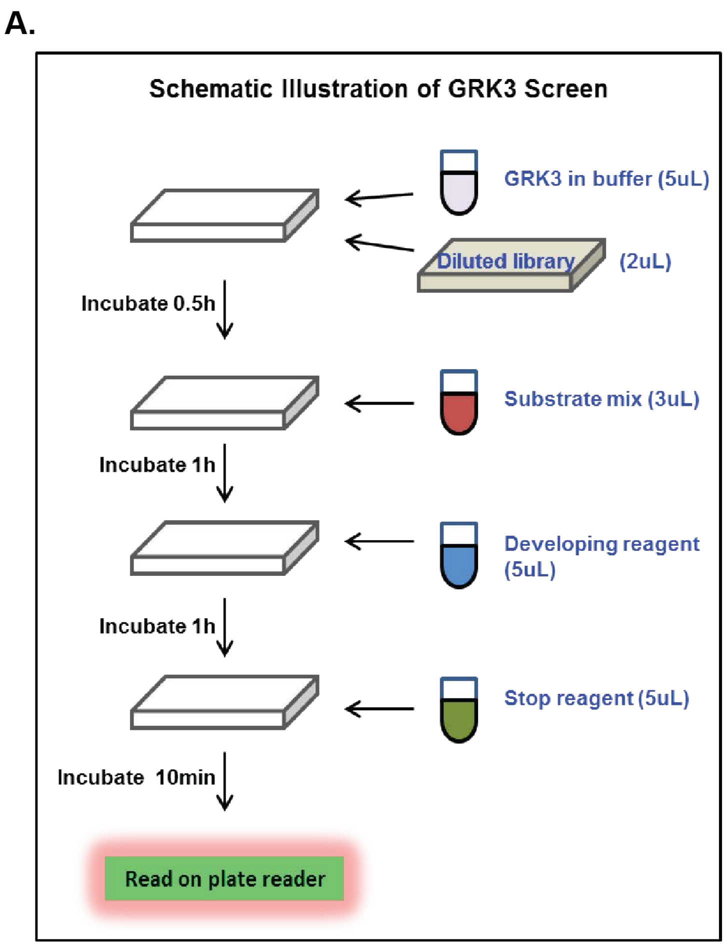

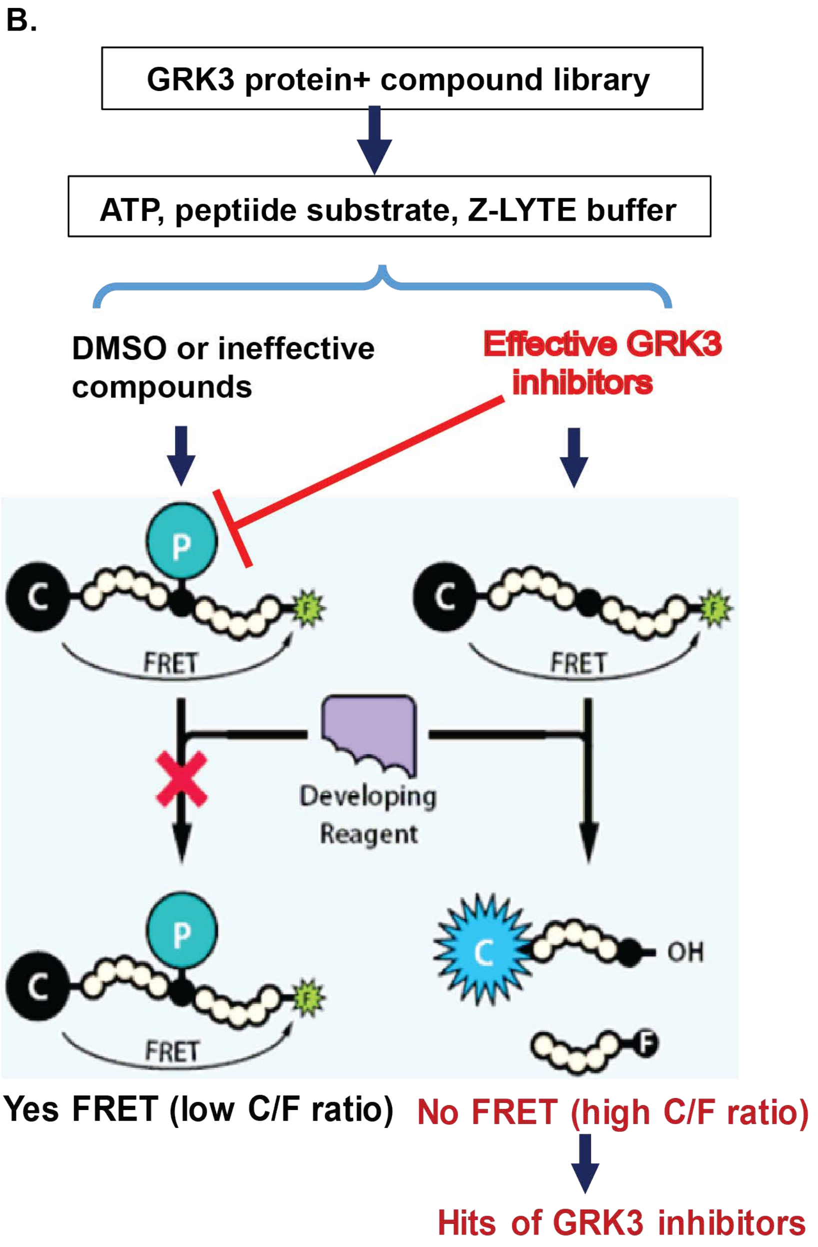

Supplementary Figure 4.

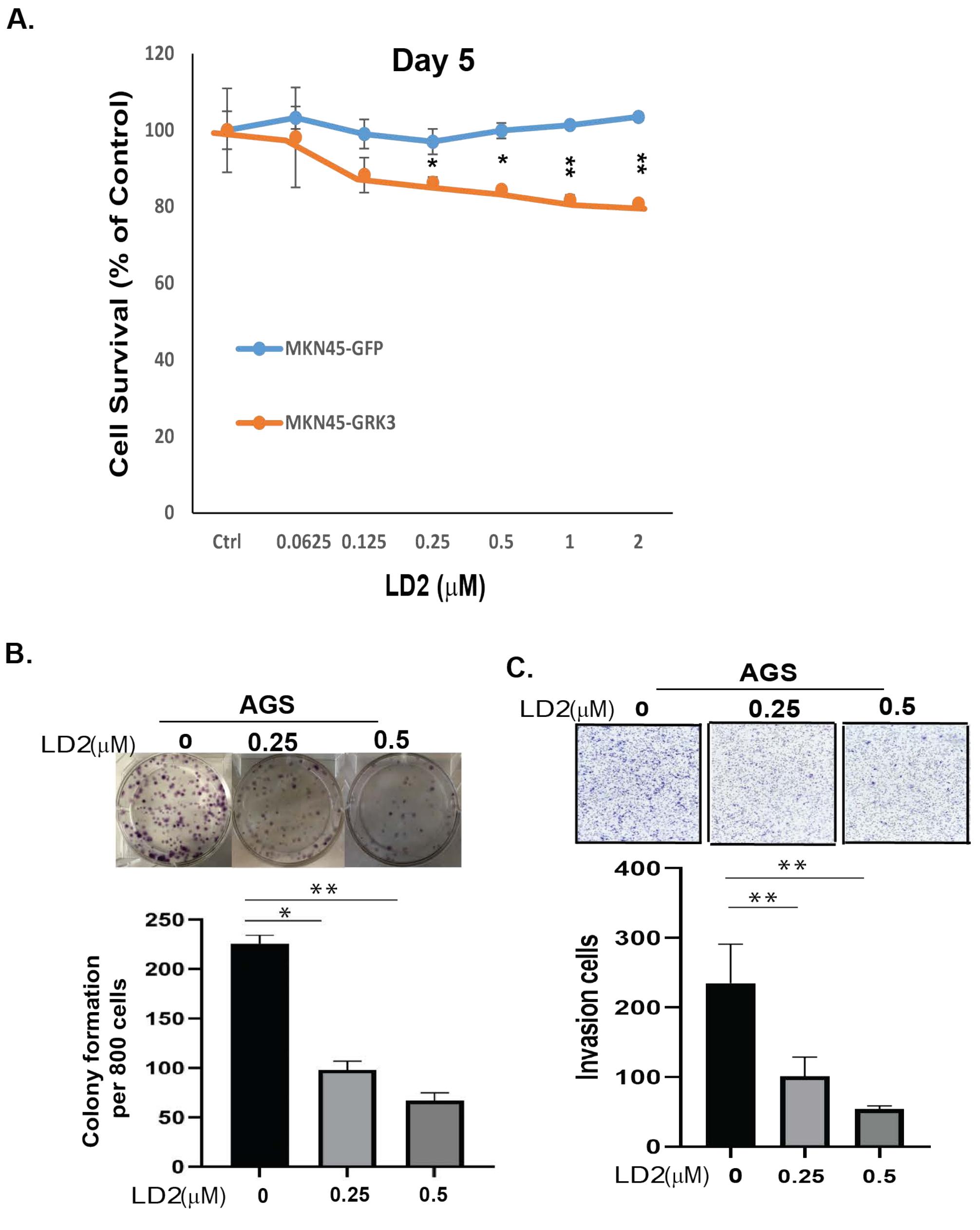

Supplementary Figure 5.

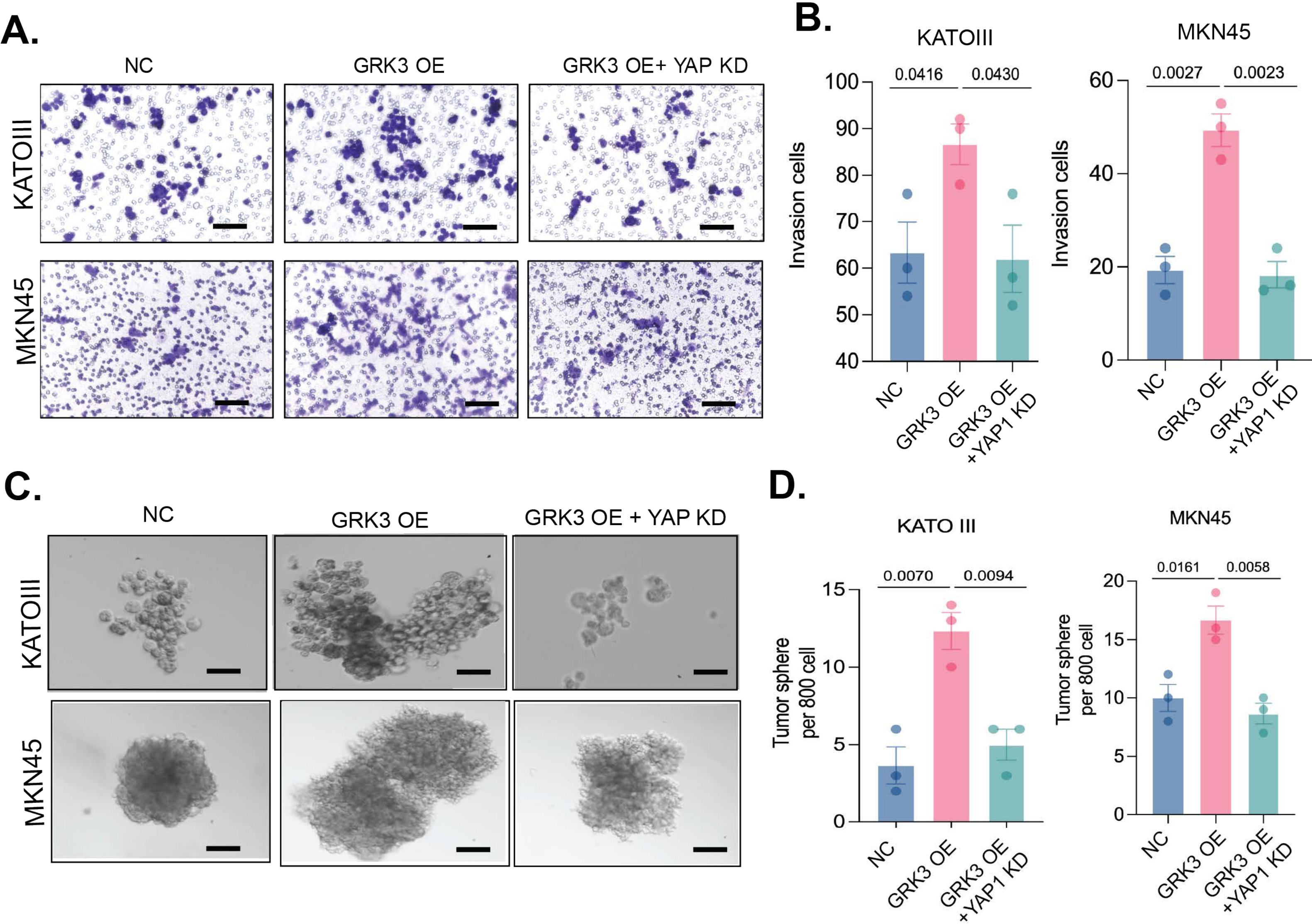

Supplementary Figure 6.

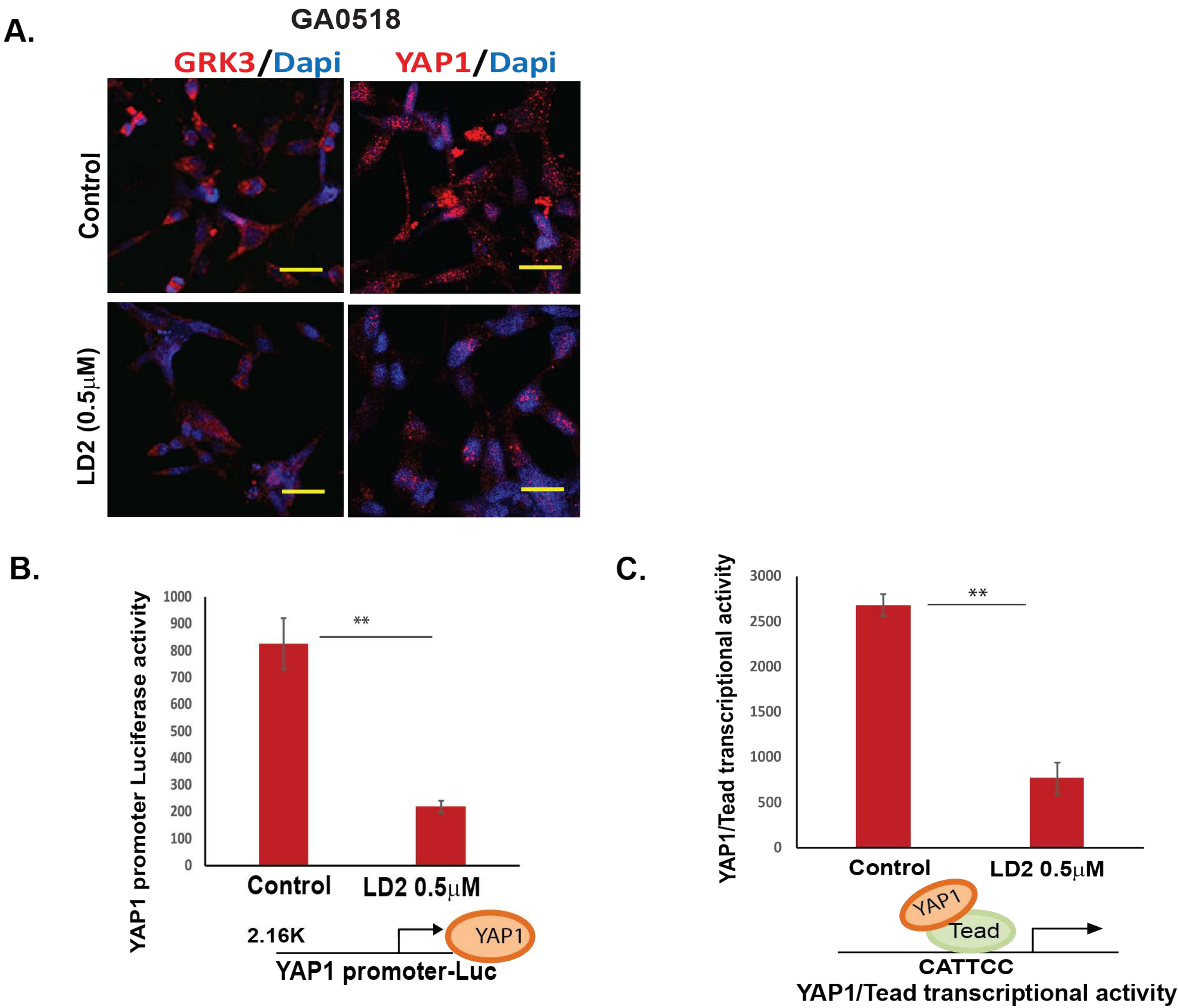

Supplementary Figure 7

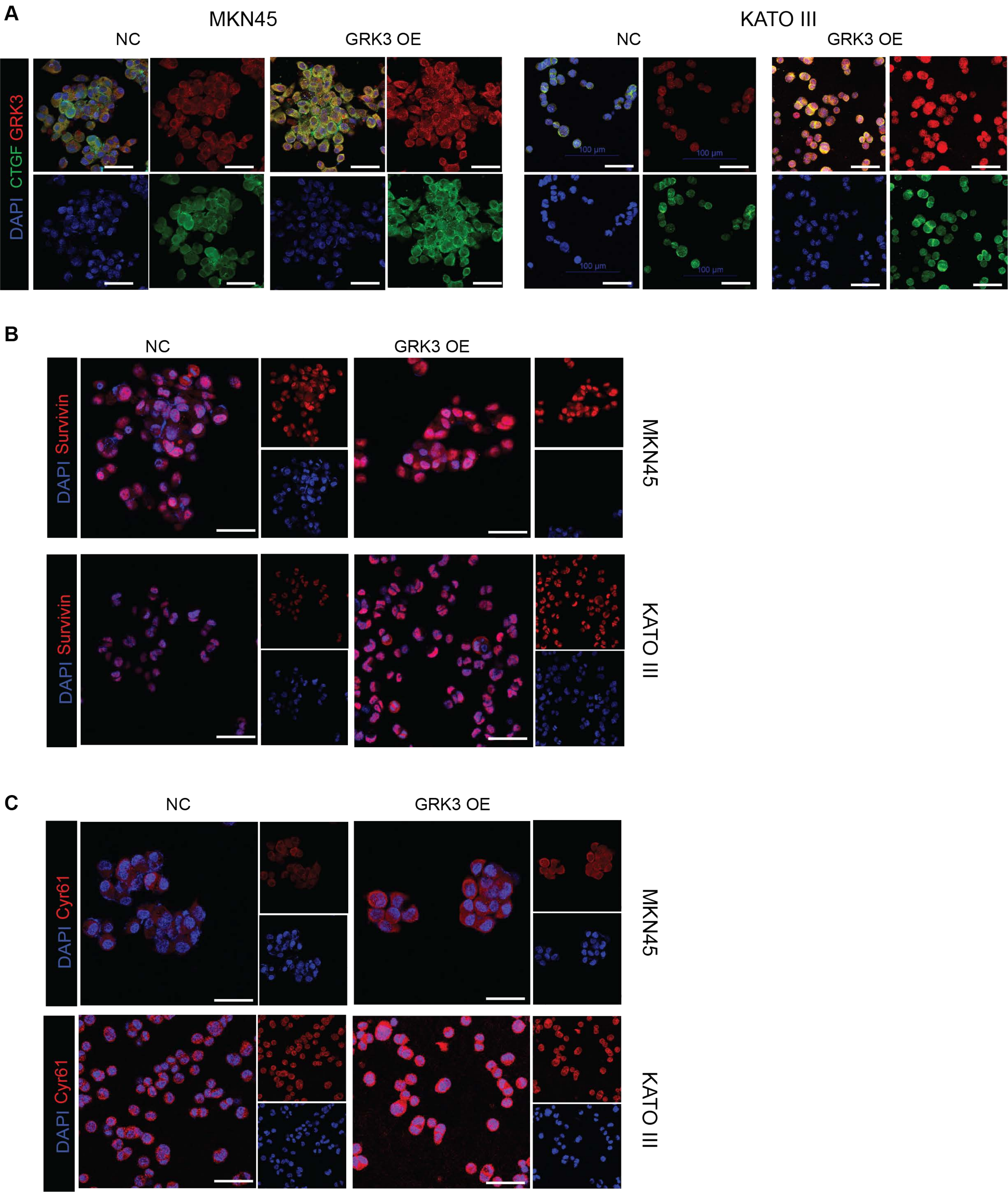

Supplementary Figure 8.

A.

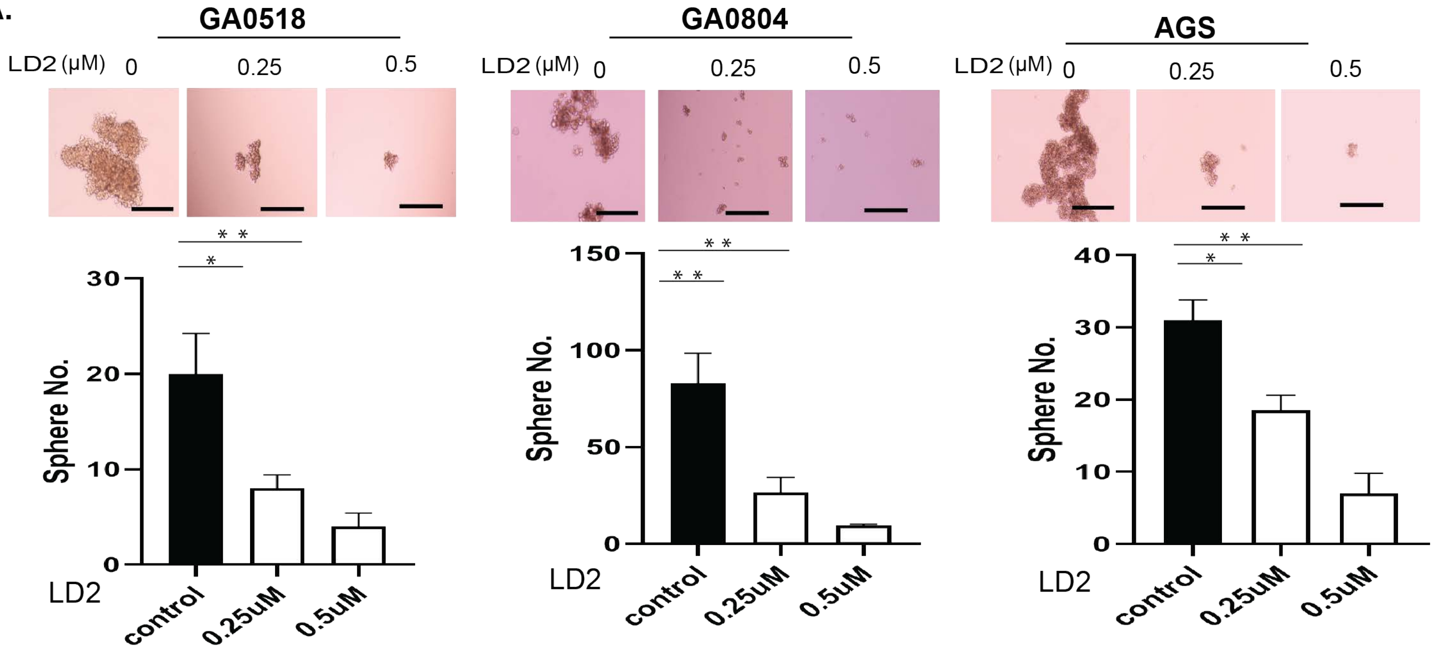

B.

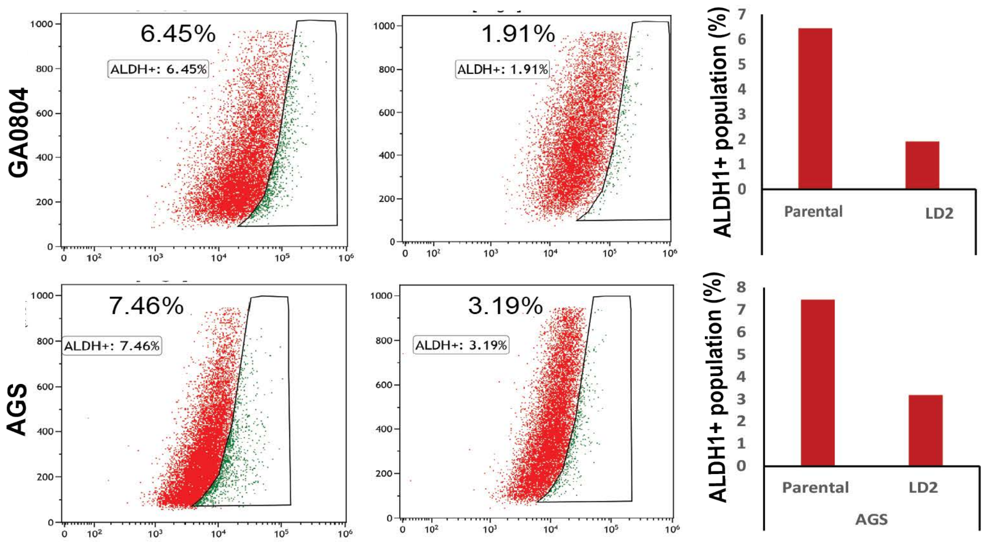

Supplementary Figure 9.

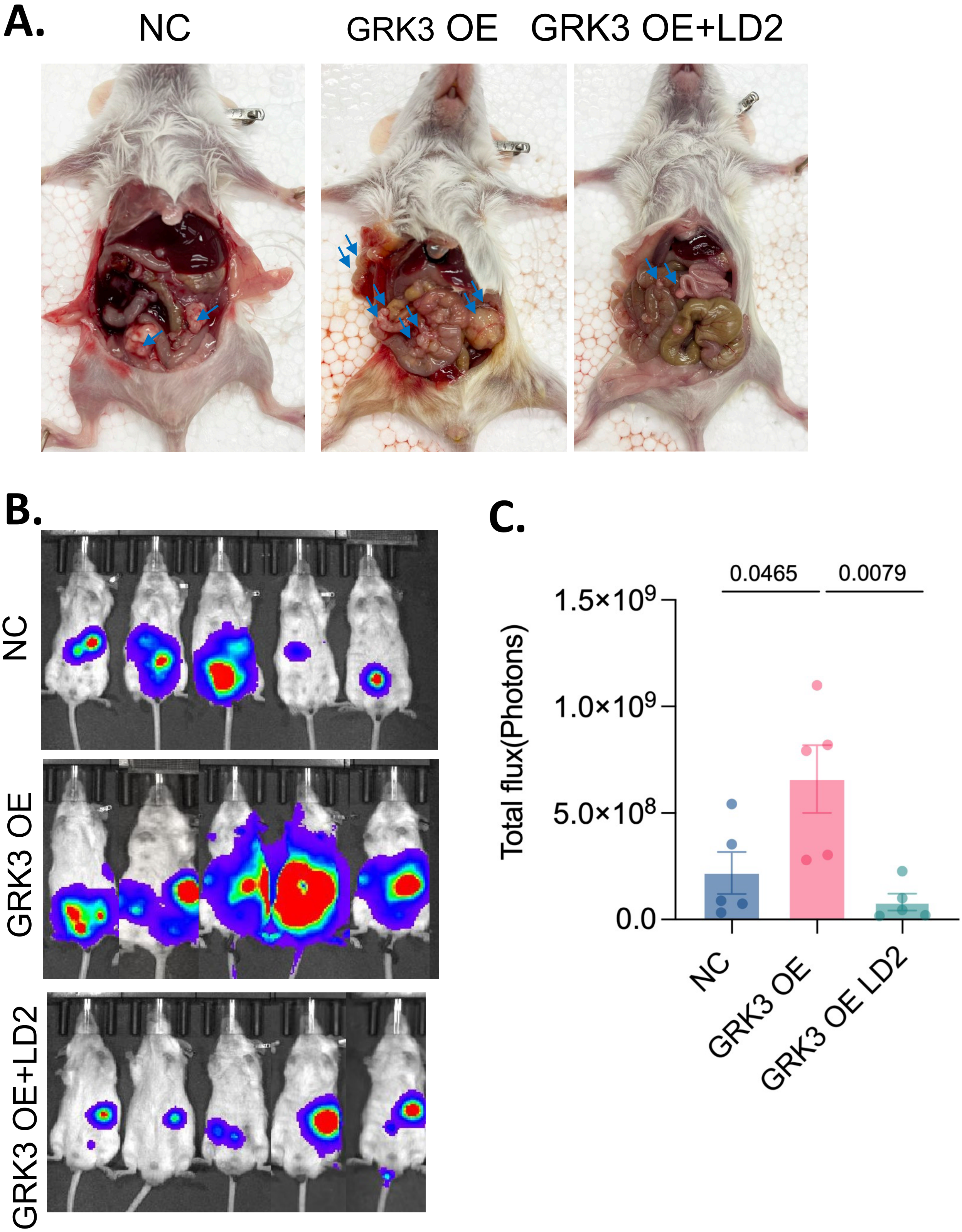

Supplement: Supplementary file 2 — Addiitional file 2: Supplementary Figure1. GRK3 expression in PDX tumors of GAC and paired primary tumor and PC. A. Diagram demonstrates the several patients’ PC cells (malignant ascites cells) into nude mice to generate PDXs (left). Western blot of GRK3 expression of Primary and ascites from PDX tumor generated from GAC patient (right). B. Representative co-immunofluorescent staining images of GRK3 and SOX9 expression on more PC specimen. Scale bars, 25µm. C. Representative co-immunofluorescent staining images of GRK3 and SOX9 expression on paired human primary tumor and PC samples. Scale bars, 25µm. Supplementary Figure 2. Overexpression of GRK3 in GAC cells increased malignant behaviors of GAC. A. Cell viability assay (MTS) was performed in MKN45-GRK3 and MKN45-GFP control cells. Fold increases of cell growth in day 6 and day 8 in media with the indicated FBS % was plotted on y-axis. **P<0.001. B. Tumor sphere formation assay was performed in MKN45 GFP and GRK3 OE cells (n = 3 biological replicates). *P<0.01. C. Invasion assay was performed in KATO III GFP, GRK3 OE and GRK3 Mut cells (n = 3 biological replicates) as described in Materias&Methods. *P<0.01; **P<0.001. Scale bar: 25µm. D. Colony formation assay was performed in KATO III GFP, GRK3 OE and GRK3 Mut cells (n = 3 biological replicates) as described in Materias&Methods. Scale bar: 100µm. **P<0.01; ***P<0.001. E. GFP and GRK3 OE cells of KATO III was cultured in 96-well plate, and MTS assay was performed at Day 3, 5, 7 and 9. **P<0.01; ***P<0.001. F. Tumor sphere assay was performed in NC and GRK KD cells of GA0518. G. AGS cells seeded in a 96-well plate were infected with a series of shRNA of GRK3 and scramble virus doses, from 20ul to 2.5ul per well. Cell survival and proliferation were measured by alamar blue viability assay 7 days after infection. X-axis represents the series of shRNA virus doses in the infections. Values on Y-axis are relative growth which was normalized to the scramble control shRNA [file 13046_2022_2463_MOESM2_ESM.pdf]
